# Supplementary material for: The Structure of an Infectious Human Polyomavirus and Its Interactions with Cellular Receptors
Source: Structure. 2018 Jun 5;26(6):839–847.e3. doi: 10.1016/j.str.2018.03.019 (PMC5992339; doi:10.1016/j.str.2018.03.019)
Supplement: Document S1. Figures S1–S5 and Table S1 [file mmc1.pdf]

Structure, Volume *26*

Supplemental Information

The Structure of an Infectious Human Polyomavirus and Its Interactions with Cellular Receptors

Daniel L. Hurdiss, Martin Frank, Joseph S. Snowden, Andrew Macdonald, and Neil A. Ranson

Supplemental data items

Table S1 Data collection and image processing, related to STAR Methods.

	BKV-GT1b	BKV-heparin	BKV-unliganded	BKV-reduced
Microscope	FEI Titan Krios	FEI Titan Krios	FEI Titan Krios	FEI Titan Krios
Camera	Falcon III	Falcon III	Falcon II	Falcon III
Voltage (kV)	300	300	300	300
Pixel size (Å)	1.065	1.065	1.065	1.065
Total dose (e^-/Å^2)	59	55	40	66
Number of frames	59	39	39	45
Defocus range (µm)	-0.5 to -3.0	-0.5 to -2.3	-0.5 to -3.0	-0.5 to -3.0
Micrographs	8651	6227	3992	3474
Acquisition software	FEI EPU	FEI EPU	FEI EPU	FEI EPU
Motion correction	MotionCor2	MotionCor2	MotionCor2	MotionCor2
CTF estimation	Gctf	Gctf	Gctf	Gctf
Software	Relion2.0	Relion2.0	Relion2.0	Relion2.0
Particles contributed	40,334	24,791	11,980	8,733
B-factor	-212	-206	-174	-326
Resolution (FSC 0.143)	3.4	3.6	3.8	6.5

Supplemental Figures

Figure S1, related to Figure 1

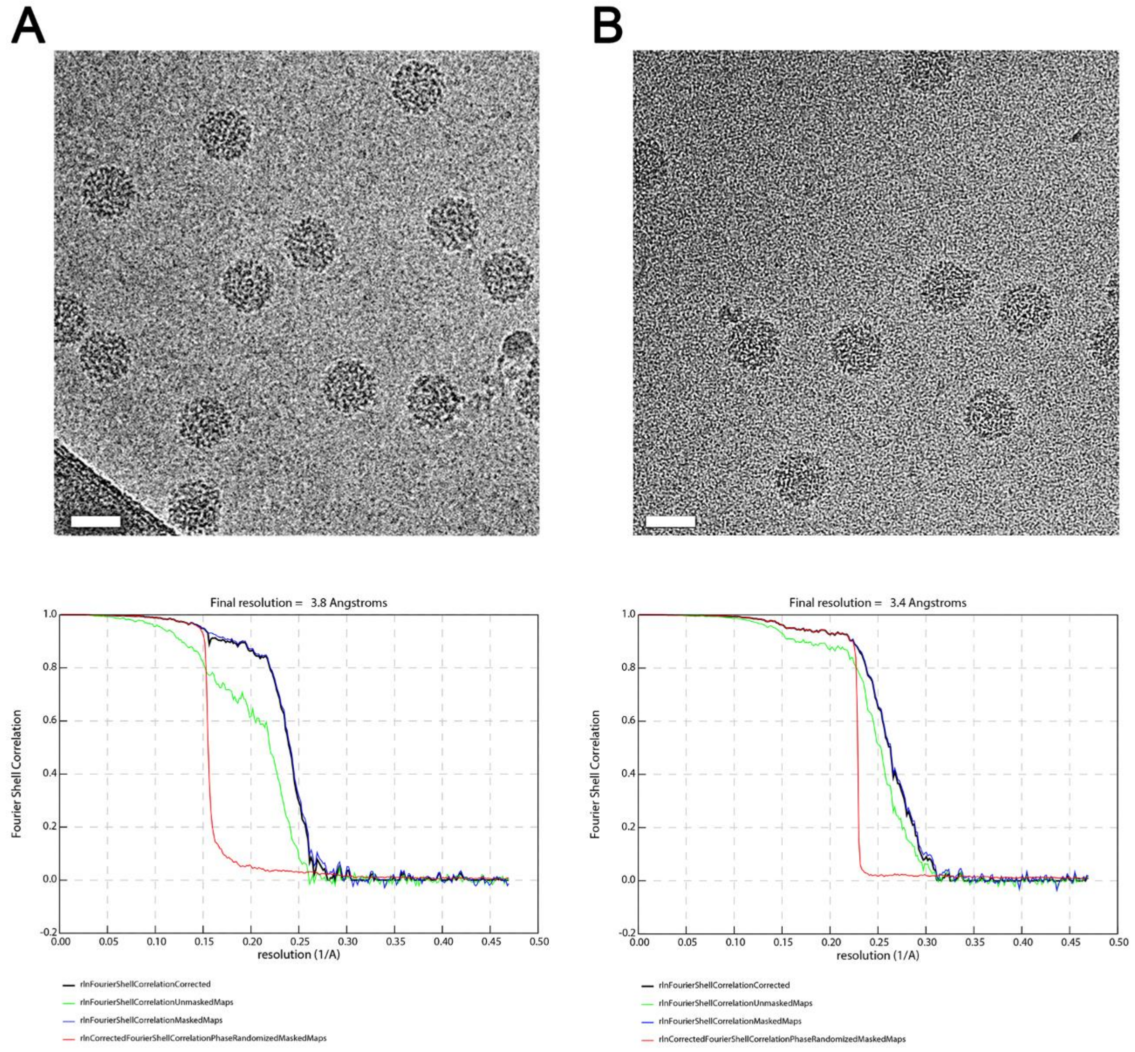

Typical micrographs from the BKV-unliganded (A) and BKV-GT1b (B) data sets (scale bar = 50 nm), with the plot of the corresponding Fourier shell correlation (FSC) below each. Based on the 0.143 criterion for the ‘gold standard’ comparison of two independent data sets, the resolution of the reconstructions is 3.8 Å (A) and 3.4 Å (B), respectively.

Figure S2, related to Figure 1

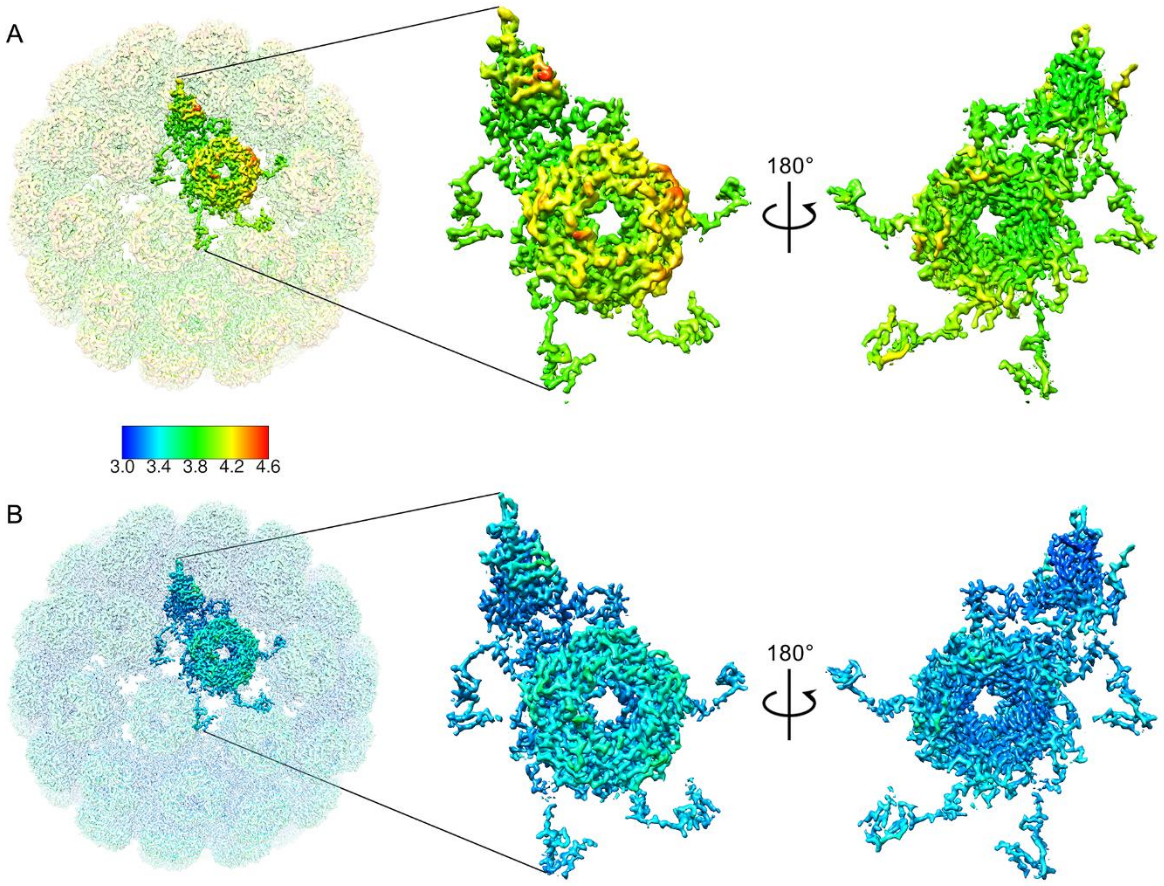

Local resolution of the BKV-unliganded (A) and BKV-GT1b (B) structures. A single asymmetric unit of BKV (seen from outside the capsid) is shown coloured according to its local resolution in both cases. The interior of the asymmetric unit is also shown by rotating the asymmetric unit 180° about the *y*-axis. The highest resolution bin is 3.0 Å (blue) and the lowest resolution bin is 4.6 Å (red). A key is shown for reference.

Figure S3, related to Figure 4

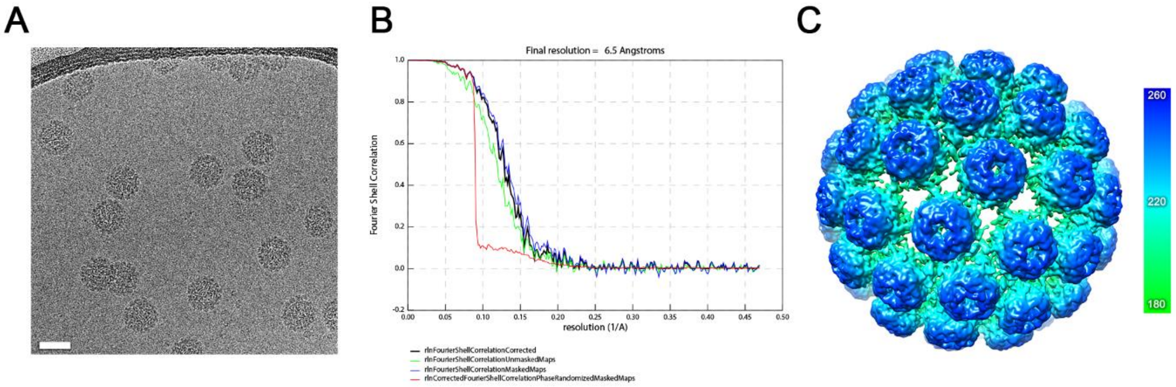

(A) Typical micrograph from the BKV-reduced data set (scale bar = 50 nm). (B) The plot of the Fourier shell correlation (FSC). Based on the 0.143 criterion for the ‘gold standard’ comparison of two independent data sets, the resolution of the reconstruction is 6.5 Å. (C) The 6.5 Å isosurface representation of reduced BKV viewed down an icosahedral two-fold axis (coloured according to the radial colouring scheme shown (Å)).

Figure S4, related to Figure 5

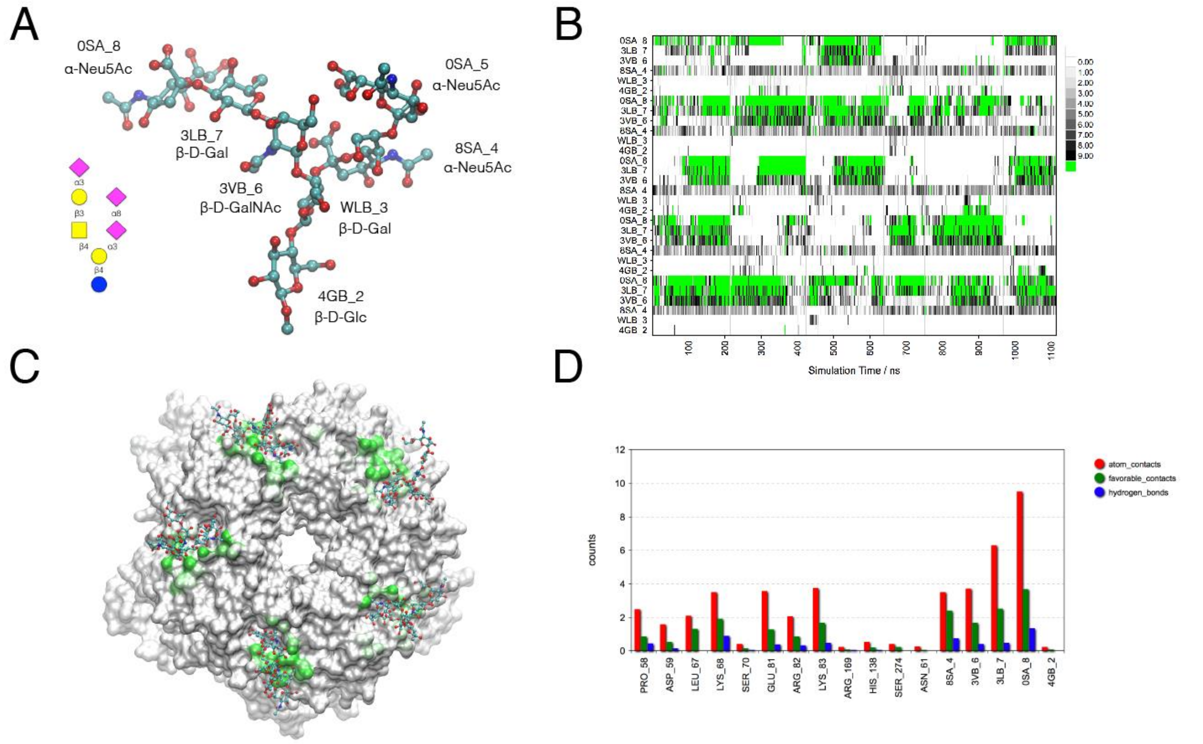

(A) SNFG-representation and 3D-model with Glycam residue labels of GT1b. (B) Trajectory of number of atom-atom contacts per residue for the five GT1B molecules. This shows the dynamics of the transient interactions. The various MD runs are separated by vertical lines. (C) Interaction 'footprint'. (D) Average number of atom-atom contacts for each residue significantly involved in binding. Note: Neu5Ac (R) (Glycam residue label 0SA_5) was excluded from the analysis.

Figure S5, related to Figure 5

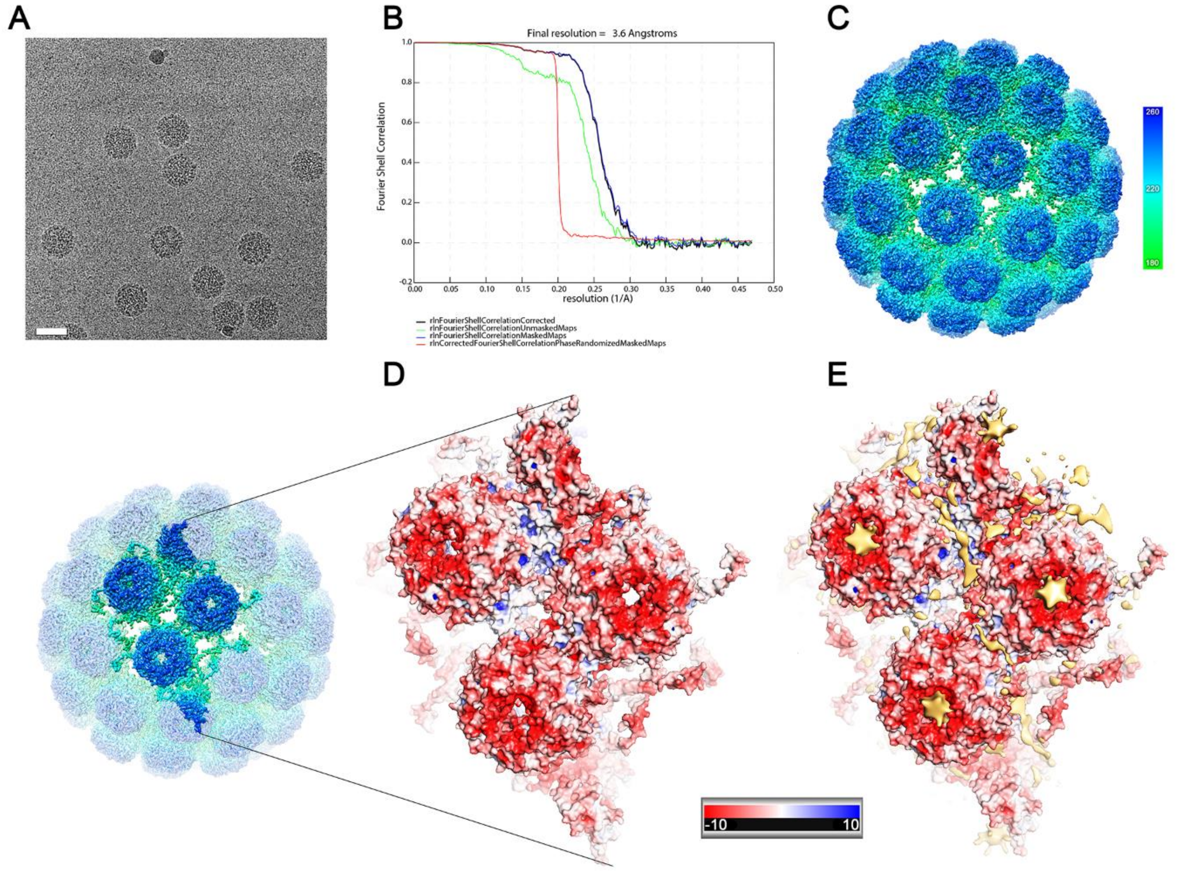

(A) Typical micrograph from the BKV-heparin data set (scale bar = 50 nm). (B) The plot of the Fourier shell correlation (FSC). Based on the 0.143 criterion for the 'gold standard' comparison of two independent data sets, the resolution of the reconstruction is 3.6 Å. (C) An isosurface representation of the 3.6 Å structure of BKV (coloured according to the radial colouring scheme shown (Å)). (D) Enlarged section of the BKV capsid with the surface colored according to electrostatic surface potential (Baker et al., 2001). (E) The same view shown in (D) overlaid with the putative heparin difference density shown in Figure 5H (orange).
