# Supplementary material for: The Structure of an Infectious Human Polyomavirus and Its Interactions with Cellular Receptors
Source: Structure. 2018 Jun 5;26(6):839–847.e3. doi: 10.1016/j.str.2018.03.019 (PMC5992339; doi:10.1016/j.str.2018.03.019)
Supplement: Document S2. Article plus Supplemental Information [file mmc2.pdf]

Structure

Article

The Structure of an Infectious Human Polyomavirus and Its Interactions with Cellular Receptors

Graphical Abstract

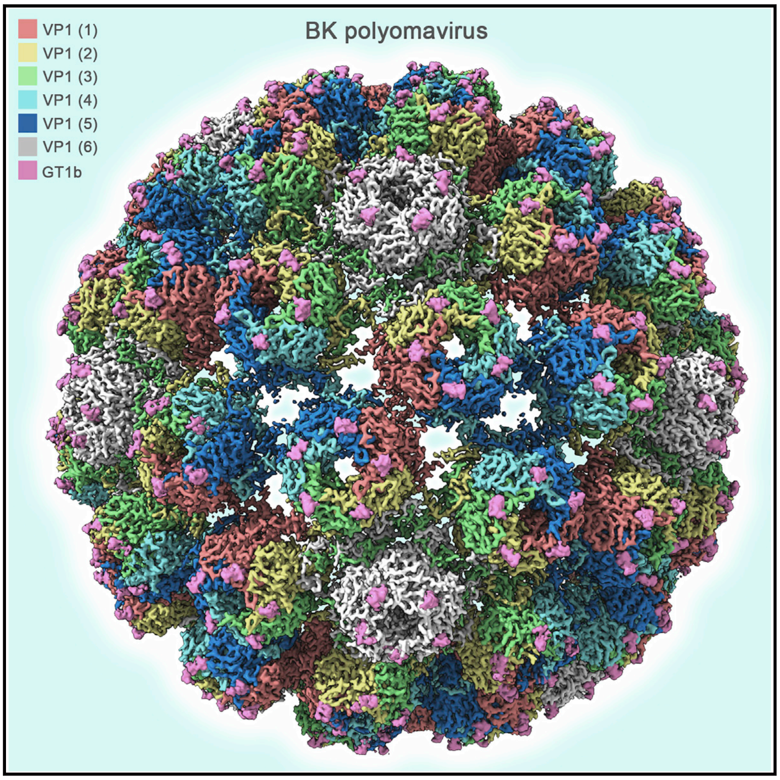

Highlights

- Present the cryo-EM structure of native, infectious BKV virion at 3.4 Å resolution
- Reveal interpentamer interactions that mediate capsid assembly
- Determine the interaction of BKV with a receptor fragment of GT1b ganglioside
- Identify possible sites for glycosaminoglycan binding on the virion surface

Authors

Daniel L. Hurdiss, Martin Frank, Joseph S. Snowden, Andrew Macdonald, Neil A. Ranson

Correspondence

a.macdonald@leeds.ac.uk (A.M.), n.a.ranson@leeds.ac.uk (N.A.R.)

In Brief

BK polyomavirus causes several debilitating diseases in the immunosuppressed with no effective treatments currently available. Hurdiss et al. present the high-resolution structure of BK and complexes with distinct cellular receptors. This work provides a platform for rational design and development of urgently needed targeted pharmacological interventions for BK-associated diseases.

Hurdiss et al., 2018, Structure *26*, 839–847
June 5, 2018 © 2018 The Authors. Published by Elsevier Ltd.
https://doi.org/10.1016/j.str.2018.03.019

The Structure of an Infectious Human Polyomavirus and Its Interactions with Cellular Receptors

Daniel L. Hurdiss,[1] Martin Frank,[2] Joseph S. Snowden,[1] Andrew Macdonald,[1,*] and Neil A. Ranson[1,3,*]
[1]Astbury Centre for Structural Molecular Biology, School of Molecular & Cellular Biology, Faculty of Biological Sciences, University of Leeds, Leeds LS2 9JT, UK
[2]Biognos AB, P.O. Box 8963, Gothenburg 40274, Sweden
[3]Lead Contact
*Correspondence: a.macdonald@leeds.ac.uk (A.M.), n.a.ranson@leeds.ac.uk (N.A.R.)
https://doi.org/10.1016/j.str.2018.03.019

SUMMARY

BK polyomavirus (BKV) causes polyomavirus-associated nephropathy and hemorrhagic cystitis in immunosuppressed patients. These are diseases for which we currently have limited treatment options, but potential therapies could include pre-transplant vaccination with a multivalent BKV vaccine or therapeutics which inhibit capsid assembly or block attachment and entry into target cells. A useful tool in such efforts would be a high-resolution structure of the infectious BKV virion and how this interacts with its full repertoire of cellular receptors. We present the 3.4-Å cryoelectron microscopy structure of native, infectious BKV in complex with the receptor fragment of GT1b ganglioside. We also present structural evidence that BKV can utilize glycosaminoglycans as attachment receptors. This work highlights features that underpin capsid stability and provides a platform for rational design and development of urgently needed pharmacological interventions for BKV-associated diseases.

INTRODUCTION

BK polyomavirus (BKV) is an opportunistic pathogen that causes severe diseases in the immunosuppressed (Bennett et al., 2012). Initial infection may occur through a respiratory or oral route, and is either asymptomatic or causes a mild respiratory illness (Goudsmit et al., 1982). A persistent infection is then established in the kidney and urinary tract, and 80%–90% of the general population are thought to be seropositive for the most prevalent BKV genotypes (Antonsson et al., 2010; Egli et al., 2009; Knowles et al., 2003; Stolt et al., 2003). In patients undergoing immunosuppression following kidney or bone marrow transplantation, lytic replication can occur, causing polyomavirus-associated nephropathy (PVAN) and hemorrhagic cystitis (Moens et al., 2017; Bennett et al., 2012; Ambalathingal et al., 2017). Up to 10% of kidney transplant patients experience PVAN, with as many as 90% of these eventually losing their graft (Ramos et al., 2009). The prevalence of BKV-associated disease is rising as the number of transplants increases (Bennett et al., 2012), and there is also mounting evidence that BKV, similar to Merkel cell polyomavirus, may be carcinogenic in humans (Müller et al., 2017; Liu et al., 2017; Papadimitriou et al., 2016; Luo et al., 2017; Frascà et al., 2015; Tillou and Doerfler, 2014; Feng et al., 2008). No effective antivirals specifically targeting BKV are available (Bennett et al., 2012; Ambalathingal et al., 2017), and so there is increasing interest in the development of targeted therapeutics. High-resolution structural information for the infectious BKV virion and its interaction with cellular receptors would be a useful resource in designing and evaluating such therapies.

Our current understanding of polyomavirus structure comes largely from pioneering work by Caspar (Griffith et al., 1992; Rayment et al., 1982) and Harrison (Stehle et al., 1996; Stehle and Harrison, 1996; Liddington et al., 1991) on SV40 (which infects Rhesus monkeys), and murine polyomavirus (PyV). Crystal structures revealed that polyomavirus capsids consist of 360 copies of the major capsid protein VP1, which form 72 "pentons," each consisting of a ring of five β barrel-containing VP1 monomers (Nilsson et al., 2005). Together, these form a $T = 7d$ lattice, with a C-terminal arm from each VP1 making interactions with neighboring pentons to stabilize the capsid shell. Essentially, polyomaviruses position pentameric capsomers in what would be the pentavalent and hexavalent positions of a true quasi-equivalent lattice (Harrison, 2017; Caspar and Klug, 1962). The asymmetric unit of these capsids therefore comprises six quasi-equivalent VP1 chains, five of which form the pentons in a hexavalent position, with the remaining VP1 chain from five asymmetric units occupying a pentavalent position. A single copy of the minor capsid protein VP2, or its N-terminal truncated variant VP3, is incorporated within each penton (Chen et al., 1998; Hurdiss et al., 2016).

Subsequently, there has been a shift toward using isolated VP1 pentons for both structural and functional studies. Binding experiments using such pentons, which cannot assemble into virus-like particles (VLPs), indicated that binding of free pentons to cells requires the presence of sialylated glycans on the host cell membrane (Maginnis et al., 2013; Neu et al., 2013a). Crystallographic structures of putative receptor ligands soaked into penton crystals have revealed the molecular mechanisms of receptor recognition (Neu et al., 2010, 2012, 2013a, 2013b; Stehle and Harrison, 1997). However, the major BKV receptors GD1b and GT1b (Low et al., 2006) cannot be soaked into crystals, presumably as a result of steric restrictions (Neu et al., 2013a). Furthermore, crystallization of isolated pentons requires the removal of the N and C termini of VP1. Therefore, such structures do not

This is an open access article under the CC BY license (http://creativecommons.org/licenses/by/4.0/).

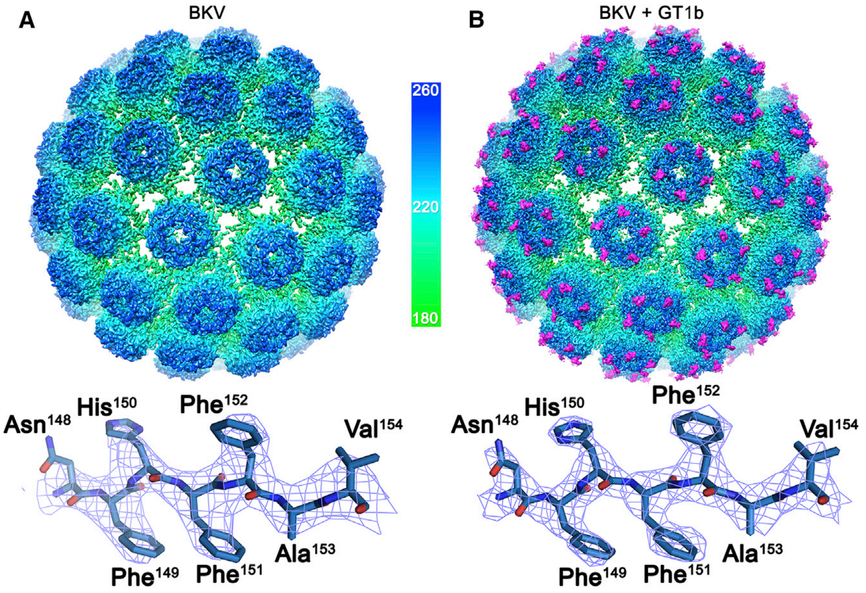

Figure 1. The Structures of BKV and BKV:GT1b

(A) Isosurface representation of the 3.8 Å structure of BKV viewed down the icosahedral 2-fold and colored according to the radial coloring scheme shown.

(B) Isosurface representation of the 3.4 Å structure of BKV, in complex with GT1b (magenta). Representative EM density containing the refined atomic model is shown below each respective structure to highlight the quality of the maps.

allow us to understand how receptors are engaged by the native virion, and penton structures may not capture the full range of surface features which could be targeted by therapeutics. Indeed, recent reports suggest BKV VLPs, but not pentons, can use glycosaminoglycans (GAGs) to attach to target cells (Geoghegan et al., 2017), suggesting that GAGs bind in regions where different capsomers interact, which are missing from capsomer structures. Cryoelectron microscopy (cryo-EM) is potentially well suited to determining the structures of intact virion: receptor complexes, but no EM structure to date has sufficient resolution to elucidate the interactions stabilizing the polyomavirus capsid, or to identify bound ligands (current structures range from 8 to 25 Å [Hurdiss et al., 2016; Li et al., 2015; Nilsson et al., 2005; Shen et al., 2011; Griffith et al., 1992]).

Here, we use high-resolution cryo-EM to determine the structure of BKV at 3.8 Å resolution. This resolution allows us to highlight differences between this human pathogen and those that infect simian and murine hosts, which include differences in how both the C-terminal arms and disulfide bonding stabilize the capsid. The importance of disulfide bonds is confirmed by a lower-resolution structure of BKV in reducing conditions in which the density we ascribe to disulfide bonds disappears. We also use a simple method analogous to crystallographic soaking, to determine the structures of BKV bound to receptors: the oligosaccharide moiety of ganglioside GT1b (at 3.4 Å resolution) and the model GAG heparin (at 3.6 Å resolution). Neither receptor causes a conformational change in the capsid, presumably functioning cooperatively to increase the avidity of attachment to the host cell surface.

RESULTS

The High-Resolution Structure of BKV:Receptor Complexes

BKV is difficult to concentrate to the levels required for optimal cryo-EM data collection, so to increase the particle concentration and distribution of BKV for cryo-EM studies, multiple aliquots of purified virions were applied to lacey carbon grids overlaid with a layer of continuous carbon (Figure S1A). Owing to the low concentration of our virus samples and low affinity of polyomavirus: receptor interactions (~1–5 mM) (Neu et al., 2008; Stehle and Harrison, 1996), to study the BKV:GT1b structure we implemented an on-grid soaking protocol, where a 20 mM solution of the oligosaccharide portion of GT1b was applied to the carbon-immobilized virions for 30 s prior to blotting and plunge freezing (Figure S1B). Collectively, these approaches allowed us to determine the structure of native BKV at 3.8 Å resolution (Figures 1A and S1A), and in complex with the oligosaccharide moiety of GT1b at 3.4 Å resolution (Figures 1B and S1B). The BKV and BKV:GT1b structures are essentially identical, other than the presence of extra, high-resolution density on the capsid surface corresponding to the oligosaccharide portion of GT1b (see below).

As expected, the BKV capsid has a $T = 7d$ quasi-symmetry, and is built from 72 pentons of the major capsid protein VP1 (Figure 2A). Each of the six distinct conformations of VP1 present within the asymmetric unit of the capsid shares a common core fold (Figures 2B/2C), which is the β sandwich, "jelly roll" fold, characteristic of many viral capsid proteins. The C-terminal arms, which invade adjacent capsomers to build the capsid, are well-resolved, and at a slightly lower resolution than the VP1 core (on average ~0.2 Å poorer; Figure S2). The resolution of our map was sufficient to identify part of the shared C terminus of VP2 and VP3, corresponding to residues 288–301 in the VP2 sequence (Figures 2D and 2E). While all pentons contain symmetry-averaged density for VP2/3 (Hurdiss et al., 2016), only the pentavalent capsomeres contained density into which an atomic model could be built. This density is located at the base of the pentons as described previously for an isolated VP1 penton from PyV (Chen et al., 1998), and describes a small hydrophobic helix that is highly conserved in the VP2/3 sequences of polyomaviruses. The polypeptide sequence for this modeled region differs by four residues between BKV and PyV, one of which localizes to the hydrophobic helix (Chen et al., 1998). Unlike PyV, there is much less interpretable density toward the N terminus of this region for BKV, perhaps reflecting increased mobility in this solution structure of the intact virion. Beneath the VP2 density (in both BKV and BKV:GT1b structures), we see the same shells of density reported at lower resolution (Hurdiss et al., 2016), but these disappear with map

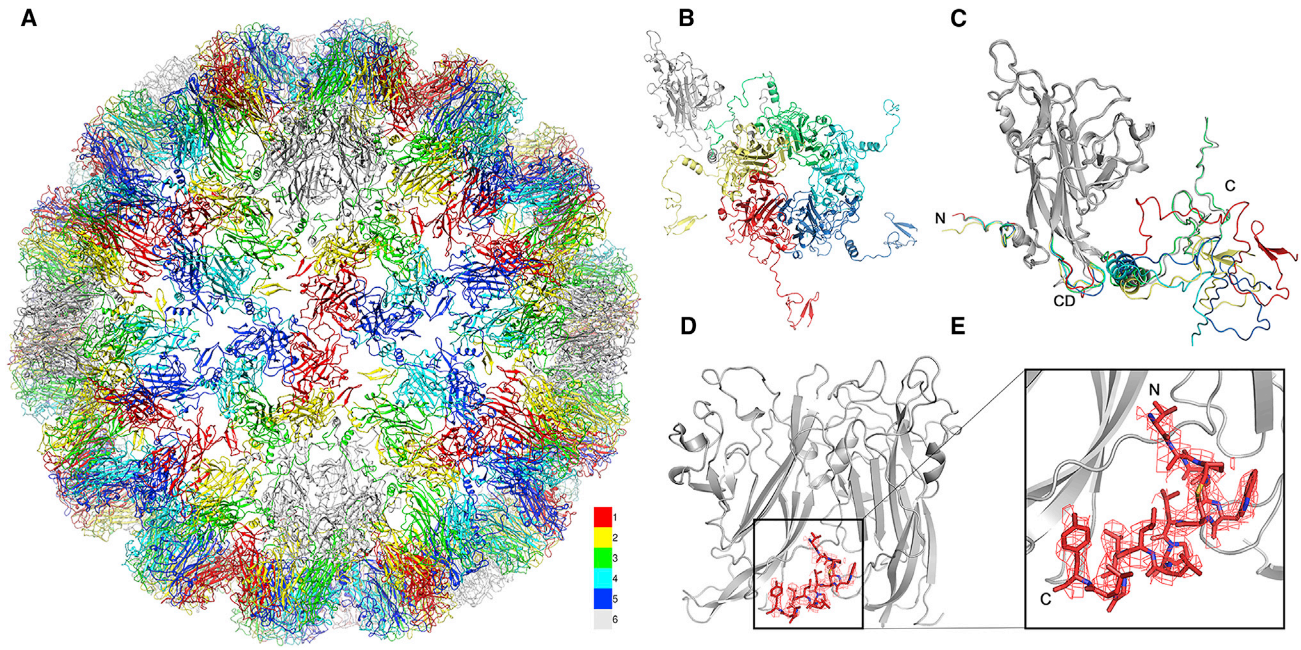

Figure 2. Atomic Model for the BKV Capsid and Interaction with the Minor Capsid Proteins

(A) The T = 7d icosahedral model of the BKV capsid viewed down an icosahedral 2-fold axis with each quasi-equivalent VP1 chain colored according to the color key shown.

(B) A single asymmetric unit of the capsid showing the conformation of each of the C termini.

(C) Overlay of all the VP1 monomers in the asymmetric unit with the most variable regions (N terminus, C terminus, and CD loop) colored according to chain number.

(D) Internal view of a 5-fold penton showing the model and EM density (2.2 σ within 1.6 Å of fitted coordinates) for the C-terminal region of VP2/VP3 binding at the base of the two adjacent VP1 monomers (gray).

(E) Enlarged view of VP2/VP3 as shown in (D).

sharpening, suggesting no high-resolution symmetric structure is present for the packaged DNA.

Structural Comparison of BKV to the Non-human Polyomavirus SV40

BKV and SV40 share 81.7% sequence identity in their major capsid protein, VP1, so, as expected, the core fold of VP1 is very similar in our BKV structure to that previously seen in SV40. However, we were able to visualize differences in the C-terminal arms of three of the six distinct conformers of VP1.

The biggest change is in chain 6 (gray in Figures 3A and 3B). In SV40, the electron density is only interpretable to residue Gln^{355}, where the polypeptide backbone points away from the virion (i.e., into bulk solution). In BKV, the polypeptide chain adopts a different conformation from Tyr^{349} onward, and in the EM density map this region is well resolved, allowing us to build an additional four residues (up to Lys^{359}). This segment is resolved in an extended conformation that makes a series of interactions with an arched structure formed by the C terminus of chain 3 (green in Figure 3C). This is a significant difference with respect to the conformation seen for SV40, as the conformation reported here is consistent with the formation of up to ten potential additional H-bonding interactions.

Differences between BKV and SV40 are also observed at the icosahedral 2-fold axes, which are formed at the interface between two adjacent chain 1s, in the region of residues 296–315 (Figure 3D). In BKV, the EM density is well resolved at the 2-fold axis, allowing us to build a conformation of the chain 1 polypeptide that forms an intimate hydrophobic interface around which the 2-fold axis of the particle is constructed (Figure 3E). This different conformation also results in a possible intramolecular interaction in BKV between Arg^{214} (Lys in SV40) in the VP1 core and Asn^{310} in the C-terminal arm (Figures 3F and 3G).

Location of Disulfide Bonds and Ca^{2+} Binding within the BKV Capsid

Polyomavirus capsids are stabilized by Ca^{2+} binding (Nilsson et al., 2005), and two putative locations for Ca^{2+} binding have been previously identified by the presence of gadolinium ions in heavy metal soaking experiments for crystallographic structure determination of SV40 (Liddington et al., 1991). In our solution structures of BKV, we see a single density consistent with a Ca^{2+} ion coordinated by glutamate side chains and the backbone carbonyl of a serine residue (Figure 4A). This location is consistent with the proposed site 1 in SV40. However, no equivalent density for any metal ion binding at site 2 is present in any of our structures.

Disulfide bond formation is also critical for capsid stability (Nilsson et al., 2005), and their reduction/isomerization in the endoplasmic reticulum of an infected host cell appears to be an essential step in virion uncoating (Schelhaas et al., 2007; Jiang et al., 2009). It has been shown previously that intermolecular disulfide bonds between Cys^{104} residues serve to stabilize the SV40 capsid, and it has been suggested, but not shown, that Cys^{9}

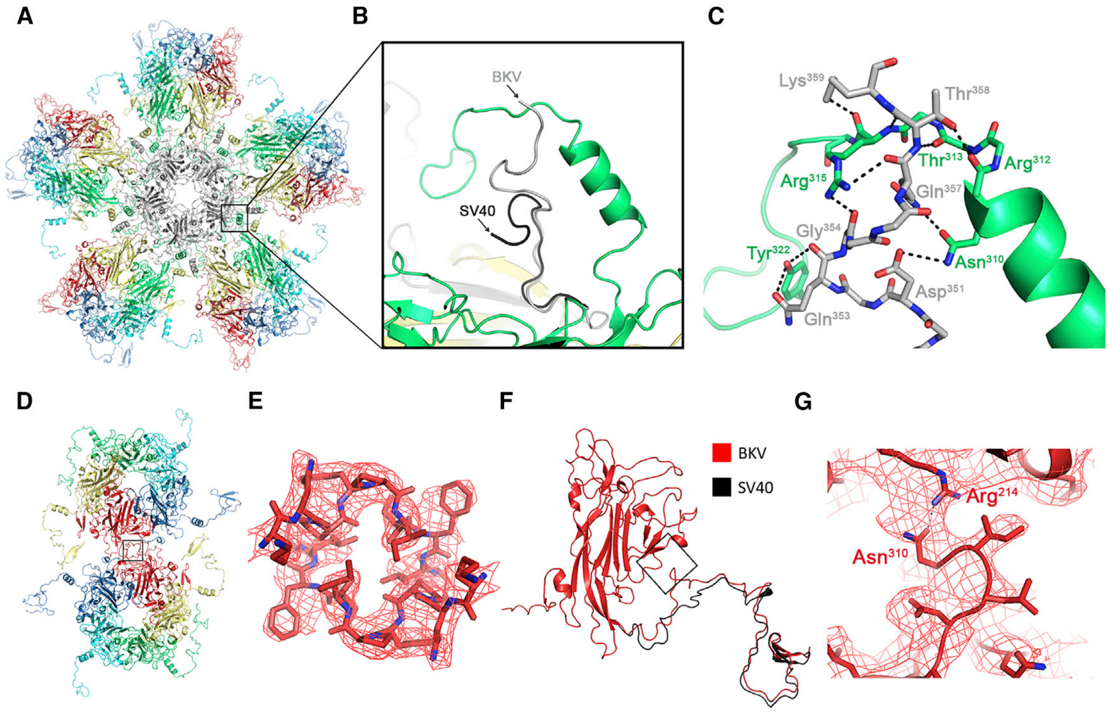

Figure 3. The C Terminus of BKV

(A) Atomic model of the 5-fold coordinated pentamer colored as described in Figure 2.

(B) Enlarged view of the C terminus of chain 6 with the equivalent region from SV40 colored black.

(C) Enlarged view of the extended conformation shown in (B) showing potential hydrogen bonds between the C terminus of chains 6 and 3 of BKV.

(D) Atomic model of two adjacent hexavalent pentamers which make up the icosahedral 2-fold.

(E) The symmetry-related C termini of chain 1 indicated by a box in (D), showing the hydrophobic interaction between residues 296–304 with EM density for the sharpened map (3 σ).

(F) A single VP1 monomer of chain 1 for BKV showing the equivalent C terminus of SV40 (black).

(G) Enlarged view of the possible intra-chain interaction denoted by a box in (F) showing EM density for the sharpened map low-pass filtered to 4 Å (2 σ).

may also be involved in disulfide bonding. The presence of Cys residues at position 104 is conserved between BKV and SV40, but the conformations of the CD loops that contain Cys^{104} are different. In SV40, the CD loops of chains 2, 3, and 6 were observed in close proximity at an interface between pentavalent and hexavalent pentons, but that SV40 structure was not able to resolve which pair of chains participated in a disulfide bond (Stehle et al., 1996). In BKV, the conformation of the CD loops is different, and strongly suggests that a disulfide bond is formed between the CD loops of chains 3 (green) and 6 (gray). The CD loop of chain 2 (yellow) is orientated away from this interaction (Figure 4B). In contrast to SV40, in BKV the N terminus of a chain 2 from an adjacent capsomere points toward the chain 2 CD loop. In our sharpened map, we are not able to resolve density for the first 13 residues of chain 2, suggesting this portion of the capsid is less well ordered. However, in the unsharpened map, which retains lower-resolution information, a bridge of density joins the N terminus to the CD loop before turning and continuing toward the encapsidated genome (Figure 4C). This density is entirely consistent with a Cys^{104}-Cys^{9} disulfide bond. A similar bridge of density is observed beneath the icosahedral 2-fold axes where the N terminus of chain 4 bridges to the CD loop of chain 1 (Figure 4E). As there is no high-resolution density for these interactions, we sought to confirm the presence of Cys^{104}-Cys^{9} disulfide bonds by determining the structure of BKV under reducing conditions (Figures S3A–S3C). The resulting structure is less ordered and has poorer resolution (6.5 Å) than the non-reduced structure (3.8 Å), likely reflecting the decreased rigidity of the virion. For direct comparison, the reduced and non-reduced structures were low-pass filtered to 7 Å. Crucially, the density for the Cys^{104}-Cys^{9} disulfides are completely absent in the reduced structure (Figures 4D and 4F).

The Interaction of BKV with GT1b Oligosaccharide

GT1b is a branched oligosaccharide, described previously as having "left" and "right" arms (Neu et al., 2013a) (Figure 5F). The disialic acid motif located on the right arm, of GT1b is well resolved in the 3.4 Å map (Figures 5A and 5B). The binding mode is similar to that seen previously in a structure of GD3, an oligosaccharide that lacks a left arm, bound to a BKV VP1 penton. The left arm of GT1b is poorly resolved in our structure, indicating that it is not ordered at high resolution. To examine the possible location of the GT1b left arm, we emphasized lower-resolution features in the map by applying a 5 Å low-pass filter to the 3.4 Å map. This revealed bridges of density between Asp^{59} and Lys^{83} of VP1 and the GT1b density (Figure 5C), consistent with previous mutagenesis data of these residues (D59Y and K83A), which resulted in significantly reduced BKV spread in cell

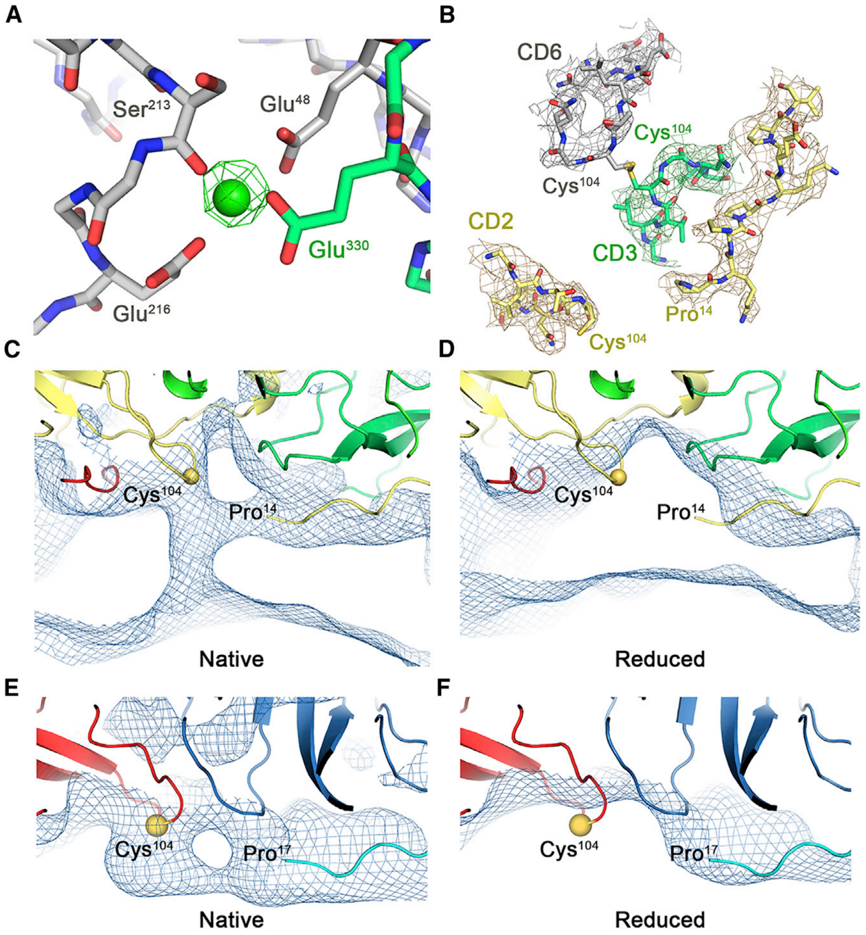

Figure 4. Location of Calcium Ions and Disulfides within the BKV Virion

(A) The calcium binding site in the sharpened BKV map with EM density for calcium shown at 6 σ with surrounding residues colored according to the corresponding chain.

(B) Atomic model of the CD loops of chains 2, 3, and 6 and the N terminus of an adjacent chain 2 from BKV with EM density shown at 1.5 σ.

(C) Density from the unsharpened, non-reduced BKV map low-pass filtered to 7 Å (0.5 σ), showing additional density extending from the N terminus of chain 2 and connecting to the CD loop of an adjacent chain 2.

(D) Analogous region to that shown in (C) from the unsharpened reduced BKV map low-pass filtered to 7 Å (0.5 σ).

(E) Density from the unsharpened BKV map low-pass filtered to 7 Å (1 σ), showing additional density extending from the N terminus of chain 4 and connecting to the CD loop of chain 1.

(F) Analogous region to that shown in (E) from the unsharpened reduced BKV map low-pass filtered to 7 Å (1 σ).

culture (Neu et al., 2013a). We also performed molecular dynamics simulations on a penton in complex with five GT1b molecules. For direct comparison with the EM density map the 3D density distribution of the GT1b heavy atoms was calculated from the accumulated 5.5 μs simulations (Figure 5D). A snapshot from this simulation reveals an orientation where Asp^{59} and Lys^{83} could interact with the left arm of GT1b (Figure 5E). Additional interactions identified during the simulation are shown in Figure S4, and it is evident that the left arm contributes to binding affinity through multiple transient interactions with the BKV surface.

To investigate recent reports of non-sialylated GAGs acting as receptors for BKV infection, we again utilized on-grid binding with the same heparin used in recently published inhibition assays (Figure S5A) (Geoghegan et al., 2017). The final sharpened map had a resolution of 3.6 Å (Figures S5B and S5C), and again there are no obvious structural differences in the BKV capsid compared with the BKV and BKV:GT1b structures. However, unlike the sharpened BKV-GT1b map, no obvious density for heparin was visible at high resolution. To identify potential low-resolution modes of binding, difference maps were calculated between the low-pass-filtered versions of the maps. The location of the resulting GT1b difference density is in agreement with our model of the branched GT1b molecule (Figure 5G), while density, which we ascribe to heparin, is located as a smear between the capsomers, and at the top of each capsomer pore (Figure 5H). The density between the capsomers locates the putative site of heparin binding atop a patch of positive charge on the virion surface (Figures S5D and S5E).

DISCUSSION

The structures of BKV presented here provide a detailed view of a native, infectious, human polyomavirus. The overall architecture of the BKV capsid is essentially the same as previously described for SV40 and PyV. However, the quality and completeness of the cryo-EM structures means that we are able to describe the different conformations of the C termini of VP1 in a particle in solution. The C termini play a fundamental role in the assembly and stability of a polyomavirus capsid, adopting six distinct conformations that allow a single VP1 sequence to adopt all of the positions in a $T = 7d$ lattice. It is unclear whether the differences we observe arise from sequence differences between SV40 and BKV or from the different solution conditions of the experiments. It should be noted, however, that the solution structures determined herein, are in more native-like conditions than those used to determine the crystal structures (Lattman, 1980; Stehle and Harrison, 1996). So, whether differences arise from experimental conditions or sequence variation, they provide insights into the structure of a human pathogen in near-native conditions. The intersubunit interactions described here (Figures 3 and 4) have implications for the structure and stability of the virus. The conformation observed for the C terminus of chain 6 (Figure 3B) suggests that it interacts with a loop of the C-terminal arm of chain 3, generating up to 10 potential H-bonding interactions (Figure 3C). Given that this interaction occurs 60 times in an intact capsid, this represents a significant source of additional stability for the BKV virion. In addition, amino acid variations between some BKV serotypes map to this region

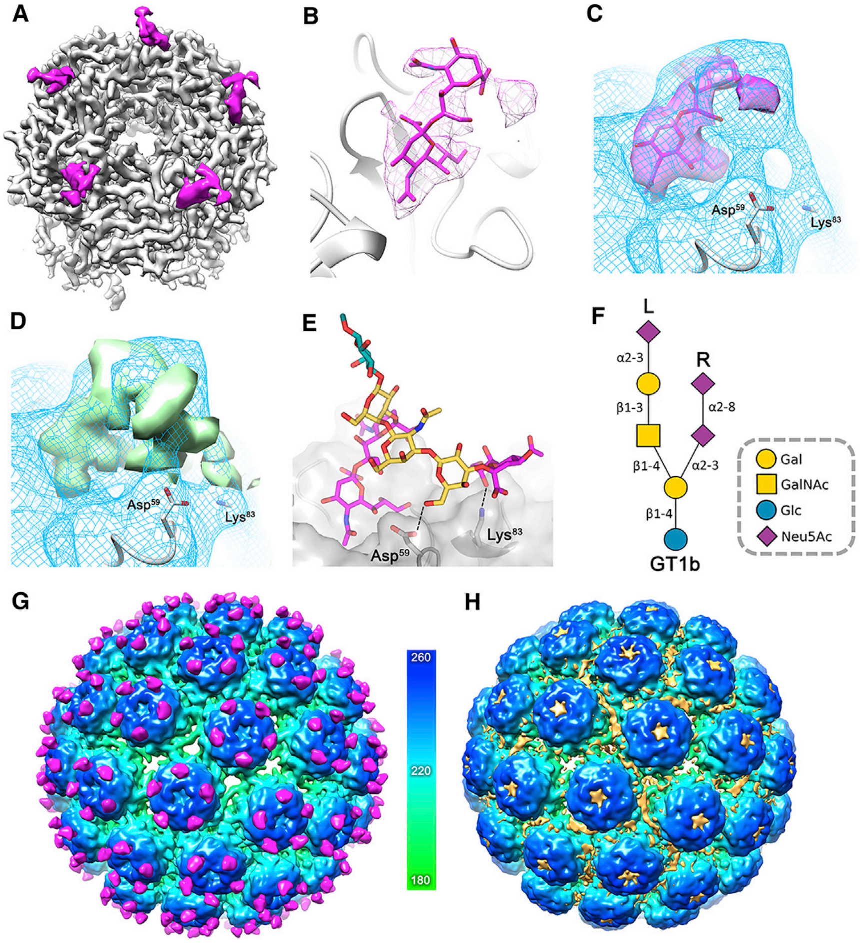

Figure 5. Interaction of BKV with GT1b and Heparin

(A) Isosurface representation of a single 5-fold pentamer of the BKV-GT1b complex with the capsid colored as in Figure 2 and GT1b (magenta).
(B) Enlarged view of the GT1b density (2 σ) containing the corresponding atomic model for the disialic acid motif of the right arm, colored as in (D).
(C) The 5 Å low-pass-filtered BKV-GT1b EM density (blue mesh) containing the high-resolution information shown in (B).
(D) MD-derived atom density map for the simulated structure of GT1b overlaid with the 5 Å low-pass-filtered EM density shown in (C).
(E) A snapshot of the MD simulated structure of GT1b showing possible interactions of the left arm with Asp^{59} and Lys^{83}.
(F) SNFG-representation of GT1b oligosaccharide showing the left and right arms of the molecule (Varki et al., 2015).
(G) Isosurface representation of the unsharpened and 8 Å low-pass-filtered BKV-GT1b map viewed down the icosahedral 2-fold axis and colored according to the radial coloring scheme shown (Å). The GT1b difference density (3.4 σ) is shown colored magenta.
(H) The unsharpened and 8 Å low-pass-filtered isosurface representation of the BKV-heparin structure, with the putative heparin difference density (3.4 σ) colored orange.

of the C terminus of VP1, indicating a possible role in antigenicity (Pastrana et al., 2013).

A different conformation is also evident in the C termini of chain 1, which make the 2-fold axes of the BKV and SV40 capsids (Figures 3D–3G), forming a tighter and more intimate hydrophobic interface. Interestingly, we see additional differences beneath (i.e., at lower radius) this interface. The N terminus of chain 4 and the CD loop of chain 1 appears to make a disulfide bond (Cys^{9}-Cys^{104}), which would further stabilize the capsid in this region. We are also able to refine the model of how pentavalent and hexavalent pentons are linked by disulfide bonds, as we show that only disulfides between Cys^{104} residues in chains 3 and 6 are present in our structure. Furthermore, previous suggestions that chain 6 might also disulfide bond to chain 2 would not be possible in our structures. The chain 2 CD loop is in a different conformation to that seen in SV40, which allows disulfide bonding to the N terminus of a second chain 2 molecule. These observations allow us to account for the disulfide bonding of all of the CD loops in the structure, and two of the N termini. We therefore present a much more complete picture of disulfide bonding in the capsid. The differences could reflect genuine differences between BKV and SV40, or indicate that the SV40 in the crystals from which the structure was solved was not in a fully oxidized state.

We also present structural data for two different receptors bound to the surface of the virus. In the 3–4 Å resolution range of the structures presented, there is no detectable conformational change in VP1. In our BKV:GT1b structure, we are able to resolve the disialic acid motif of the GT1b right arm to high resolution, confirming a conserved mode of binding between the large B-series gangliosides and smaller variants studied previously. MD simulations show that the left arm of GT1b is dynamic, explaining the lack of high-resolution density in our EM map, and its previous intractability to crystallographic studies. Low-resolution EM density and MD suggest a likely mechanism of binding for the left arm of GT1b, in which multiple, weak interactions occur with the VP1 surface. Such interactions provide the structural correlate to previous mutagenic and functional studies showing that the presence of a left arm enhances infectivity (Neu et al., 2013a).

These results demonstrate the emerging role cryo-EM can play in structural glycobiology, to complement existing nuclear magnetic resonance and crystallographic approaches. The ability to produce polyomavirus-ligand structures at resolutions approaching that of X-ray crystallography also leads to the exciting prospect that cryo-EM could be used to study small molecule inhibitors of BK infection or assembly, and indeed the structure of BKV:GT1b presented here is an ideal platform for the *in silico* design of such tool molecules. It is clear that neither GT1b nor GAG binding induce detectable conformational change in the VP1 component of the capsid, and presumably act simply as attachment receptors to facilitate cell entry, where changes in

redox state and pH can effect changes in capsid structure that lead to uncoating (Jiang et al., 2009).

It is not possible to determine whether the density between pentamers represents bound GAG molecules, but the lack of resolution suggests that no single mode of binding exists. However, this site is positively charged, as are GAG binding sites observed in other viruses (O'Donnell et al., 2009; Fry et al., 1999). Density in the pore of each pentamer and at the 2-fold axes would be subject to inappropriate symmetry averaging, and so must be interpreted with great care. The possibility that GAGs bind in the pore of the VP1 pentamer is intriguing however. We see no evidence for an interaction between GAGs and VP2/3, but the pore is directly above their location, and it is interesting to note that BKV pseudoviruses that lack VP2 and VP3 are less efficient at transducing a range of different cell types (Schowalter and Buck, 2013), which might be partially explained by a reduced affinity for GAGs. Indeed, the entire VP1 shell is remarkably porous, potentially allowing other opportunities for the interactions between minor capsid proteins and GAGs that have been suggested for a related virus, human papillomavirus 16 (Guan et al., 2017). Our observations provide structural clues about GAG binding to BKV and may form a basis to determine the precise molecular mechanism of GAG interaction using shorter, defined fragments of heparin, which may be more amenable to high-resolution structural characterization. Interestingly GAG analogs have reportedly been used to treat BKV-associated disease (Van der Aa et al., 2014; Winter et al., 2015; Isik et al., 2014; Cervigni, 2015). The rationale for such treatments was based on restoration of the barrier function of the bladder epithelium, but in light of recent results (including those presented here), it is possible that GAGs may bind directly to BKV and perturb cellular attachment or entry.

The rationale for the studies presented here is to inform the design of future anti-BKV therapies. Such therapies could include antibodies capable of neutralizing BKV, or BKV vaccination. Human mAbs which bind BKV virions have recently been reported (Jelcic et al., 2015), and vaccination with BKV VLPs has been shown to induce pan-specific immunity in mouse models (Pastrana et al., 2013). Our structures of the native, reduced, and receptor-bound virions provide a platform for understanding such antibody responses to BKV, and features that underpin vaccine stability. Going forward, co-structures with mAbs that show BKV serotype-specific neutralization (Randhawa et al., 2009), broad cross-neutralization, or non-neutralizing mAbs will be key for guiding efforts to further develop therapies to protect patients against BKV nephropathy (Buck, 2016).

STAR★METHODS

Detailed methods are provided in the online version of this paper and include the following:

- KEY RESOURCES TABLE
- CONTACT FOR REAGENT AND RESOURCE SHARING
- METHOD DETAILS
  - Cell Culture
  - Virus Growth
  - Virus Purification
  - Electron Microscopy
  - Image Processing
  - Model Building and Refinement
  - Molecular Dynamics Simulations
- QUANTIFICATION AND STATISTICAL ANALYSIS
- DATA AND SOFTWARE AVAILABILITY

SUPPLEMENTAL INFORMATION

Supplemental Information includes five figures and one table and can be found with this article online at https://doi.org/10.1016/j.str.2018.03.019.

ACKNOWLEDGMENTS

We thank the Wellcome Trust for PhD Studentship support to D.L.H. (102572/B/13/Z) and J.S.S. (102174/B/13/Z). We also thank Kidney Research UK (RP25/2013 and ST4/2014) and Yorkshire Kidney Research Fund for support. We thank Emma Hesketh for assistance with data collection (who is partly supported by BBSRC BBL021250/1). All electron microscopy was performed in the Astbury Biostructure Laboratory, which was funded by the University of Leeds and the Wellcome Trust (108466/Z/15/Z). We thank the support staff at the Astbury Biostructure Laboratory for their kind help.

AUTHOR CONTRIBUTIONS

Conceptualization, D.L.H., A.M., and N.A.R.; Methodology, D.L.H., M.F., A.M., and N.A.R.; Investigation, D.L.H., M.F., and J.S.S.; Writing – Review & Editing, D.L.H., M.F., J.S.S., A.M., and N.A.R.; Visualization, D.L.H., M.F., and N.A.R.; Supervision, A.M. and N.A.R.

DECLARATION OF INTERESTS

The authors declare no competing interests.

Received: January 18, 2018
Revised: March 14, 2018
Accepted: March 29, 2018
Published: April 26, 2018

SUPPORTING CITATIONS

The following reference appears in the Supplemental Information: Baker et al., 2001.

REFERENCES

Abend, J.R., Low, J.A., and Imperiale, M.J. (2007). Inhibitory effect of gamma interferon on BK virus gene expression and replication. J. Virol. *81*, 272–279.

Ambalathingal, G.R., Francis, R.S., Smyth, M.J., Smith, C., and Khanna, R. (2017). BK polyomavirus: clinical aspects, immune regulation, and emerging therapies. Clin. Microbiol. Rev. *30*, 503–528.

Antonsson, A., Green, A.C., Mallitt, K.-A., O'Rourke, P.K., Pawlita, M., Waterboer, T., and Neale, R.E. (2010). Prevalence and stability of antibodies to the BK and JC polyomaviruses: a long-term longitudinal study of Australians. J. Gen. Virol. *91*, 1849–1853.

Baker, N.A., Sept, D., Joseph, S., Holst, M.J., and McCammon, J.A. (2001). Electrostatics of nanosystems: application to microtubules and the ribosome. Proc. Natl. Acad. Sci. USA *98*, 10037–10041.

Bennett, S.M., Broekema, N.M., and Imperiale, M.J. (2012). BK polyomavirus: emerging pathogen. Microbes Infect. *14*, 672–683.

Buck, C.B. (2016). Exposing the molecular machinery of BK polyomavirus. Structure *24*, 495.

Caspar, D.L., and Klug, A. (1962). Physical principles in the construction of regular viruses. Cold Spring Harb. Symp. Quant. Biol. *27*, 1–24.

Cervigni, M. (2015). Interstitial cystitis/bladder pain syndrome and glycosaminoglycans replacement therapy. Transl. Androl. Urol. *4*, 638–642.

Chen, X.S., Stehle, T., and Harrison, S.C. (1998). Interaction of polyomavirus internal protein VP2 with the major capsid protein VP1 and implications for participation of VP2 in viral entry. EMBO J. *17*, 3233–3240.

Egli, A., Infanti, L., Dumoulin, A., Buser, A., Samaridis, J., Stebler, C., Gosert, R., and Hirsch, H.H. (2009). Prevalence of polyomavirus BK and JC infection and replication in 400 healthy blood donors. J. Infect. Dis. *199*, 837–846.

Emsley, P., Lohkamp, B., Scott, W.G., and Cowtan, K. (2010). Features and development of Coot. Acta Crystallogr. D Biol. Crystallogr. *66*, 486–501.

Feng, H., Shuda, M., Chang, Y., and Moore, P.S. (2008). Clonal integration of a polyomavirus in human Merkel cell carcinoma. Science *319*, 1096–1100.

Frascà, G.M., Sandrini, S., Cosmai, L., Porta, C., Asch, W., Santoni, M., Salviani, C., D'Errico, A., Malvi, D., Balestra, E., et al. (2015). Renal cancer in kidney transplanted patients. J. Nephrol. *28*, 659–668.

Fry, E.E., Lea, S.M., Jackson, T., Newman, J.W., Ellard, F.M., Blakemore, W.E., Abu-Ghazaleh, R., Samuel, A., King, A.M., and Stuart, D.I. (1999). The structure and function of a foot-and-mouth disease virus-oligosaccharide receptor complex. EMBO J. *18*, 543–554.

Geoghegan, E.M., Pastrana, D.V., Schowalter, R.M., Ray, U., Gao, W., Ho, M., Pauly, G.T., Sigano, D.M., Kaynor, C., Cahir-McFarland, E., et al. (2017). Infectious entry and neutralization of pathogenic JC polyomaviruses. Cell Rep. *21*, 1169–1179.

Goudsmit, J., Dillen, P.W., van Strien, A., and van der Noordaa, J. (1982). The role of BK virus in acute respiratory tract disease and the presence of BKV DNA in tonsils. J. Med. Virol. *10*, 91–99.

Griffith, J.P., Griffith, D.L., Rayment, I., Murakami, W.T., and Caspar, D.L. (1992). Inside polyomavirus at 25-A resolution. Nature *355*, 652–654.

Guan, J., Bywaters, S.M., Brendle, S.A., Ashley, R.E., Makhov, A.M., Conway, J.F., Christensen, N.D., and Hafenstein, S. (2017). Cryoelectron microscopy maps of human papillomavirus 16 reveal L2 densities and heparin binding site. Structure *25*, 253–263.

Harrison, S.C. (2017). Protein tentacles. J. Struct. Biol. *200*, 244–247.

Headd, J.J., Echols, N., Afonine, P.V., Grosse-Kunstleve, R.W., Chen, V.B., Moriarty, N.W., Richardson, D.C., Richardson, J.S., and Adams, P.D. (2012). Use of knowledge-based restraints in phenix.refine to improve macromolecular refinement at low resolution. Acta Crystallogr. D Biol. Crystallogr. *68*, 381–390.

Humphrey, W., Dalke, A., and Schulten, K. (1996). VMD: visual molecular dynamics. J. Mol. Graph. *14*, 33–38.

Hurdiss, D.L., Morgan, E.L., Thompson, R.F., Prescott, E.L., Panou, M.M., Macdonald, A., and Ranson, N.A. (2016). New structural insights into the genome and minor capsid proteins of BK polyomavirus using cryo-electron microscopy. Structure *24*, 528–536.

Isik, P., Özbek, N., Azik, F., Arman Bilir, Ö., Baylan, B., and Tunç, B. (2014). Successful treatment of hemorrhagic cystitis after hematopoietic stem cell transplantation with intravesical hyaluronic acid. Pediatr. Transpl. *18*, 780–781.

Jelcic, I., Combaluzier, B., Jelcic, I., Faigle, W., Senn, L., Reinhart, B.J., Ströh, L., Nitsch, R.M., Stehle, T., Sospedra, M., et al. (2015). Broadly neutralizing human monoclonal JC polyomavirus VP1-specific antibodies as candidate therapeutics for progressive multifocal leukoencephalopathy. Sci. Transl. Med. *7*, 306ra150.

Jiang, M., Abend, J.R., Tsai, B., and Imperiale, M.J. (2009). Early events during BK virus entry and disassembly. J. Virol. *83*, 1350–1358.

Kimanius, D., Forsberg, B.O., Scheres, S.H., and Lindahl, E. (2016). Accelerated cryo-EM structure determination with parallelisation using GPUs in RELION-2. Elife *5*, e18722.

Kirschner, K.N., Yongye, A.B., Tschampel, S.M., González-Outeiriño, J., Daniels, C.R., Foley, B.L., and Woods, R.J. (2008). GLYCAM06: a generalizable biomolecular force field. Carbohydrates. J. Comput. Chem. *29*, 622–655.

Knowles, W.A., Pipkin, P., Andrews, N., Vyse, A., Minor, P., Brown, D.W.G., and Miller, E. (2003). Population-based study of antibody to the human polyomaviruses BKV and JCV and the simian polyomavirus SV40. J. Med. Virol. *71*, 115–123.

Krieger, E., and Vriend, G. (2015). New ways to boost molecular dynamics simulations. J. Comput. Chem. *36*, 996–1007.

Krissinel, E., and Henrick, K. (2007). Inference of macromolecular assemblies from crystalline state. J. Mol. Biol. *372*, 774–797.

Lattman, E.E. (1980). Simian virus 40 crystals. Science *208*, 1048–1050.

Li, T.-C., Iwasaki, K., Katano, H., Kataoka, M., Nagata, N., Kobayashi, K., Mizutani, T., Takeda, N., Wakita, T., and Suzuki, T. (2015). Characterization of self-assembled virus-like particles of Merkel cell polyomavirus. PLoS One *10*, e0115646.

Liddington, R.C., Yan, Y., Moulai, J., Sahli, R., Benjamin, T.L., and Harrison, S.C. (1991). Structure of simian virus 40 at 3.8-A resolution. Nature *354*, 278–284.

Liu, S., Chaudhry, M.R., Berrebi, A.A., Papadimitriou, J.C., Drachenberg, C.B., Haririan, A., and Alexiev, B.A. (2017). Polyomavirus replication and smoking are independent risk factors for bladder cancer after renal transplantation. Transplantation *101*, 1488–1494.

Low, J.A., Magnuson, B., Tsai, B., and Imperiale, M.J. (2006). Identification of gangliosides GD1b and GT1b as receptors for BK virus. J. Virol. *80*, 1361–1366.

Luo, H.L., Chen, Y.T., Huang, S.C., Chen, T.C., Chiang, P.H., Lin, S.S., and Cheng, Y.T. (2017). Human polyomavirus is associated with earlier onset of upper urinary tract urothelial carcinoma in patients after kidney transplantation. Transplant. Proc. *49*, 1064–1067.

Maginnis, M.S., Ströh, L.J., Gee, G.V., O'Hara, B.A., Derdowski, A., Stehle, T., and Atwood, W.J. (2013). Progressive multifocal leukoencephalopathy-associated mutations in the JC polyomavirus capsid disrupt lactoseries tetrasaccharide c binding. MBio *4*, e00247–00213.

Moens, U., Krumbholz, A., Ehlers, B., Zell, R., Johne, R., Calvignac-Spencer, S., and Lauber, C. (2017). Biology, evolution, and medical importance of polyomaviruses: an update. Infect. Genet. Evol. *54*, 18–38.

Müller, D.C., Rämö, M., Naegele, K., Ribi, S., Wetterauer, C., Perrina, V., Quagliata, L., Vlajnic, T., Ruiz, C., Balitzki, B., et al. (2017). Donor-derived, metastatic urothelial cancer after kidney-transplantation associated with a potentially oncogenic BK polyomavirus. J. Pathol. *244*, 265–270.

Neu, U., Allen, S.A., Blaum, B.S., Liu, Y., Frank, M., Palma, A.S., Ströh, L.J., Feizi, T., Peters, T., Atwood, W.J., et al. (2013a). A structure-guided mutation in the major capsid protein retargets BK polyomavirus. PLOS Pathog. *9*, e1003688.

Neu, U., Hengel, H., Blaum, B.S., Schowalter, R.M., Macejak, D., Gilbert, M., Wakarchuk, W.W., Imamura, A., Ando, H., Kiso, M., et al. (2012). Structures of Merkel cell polyomavirus VP1 complexes define a sialic acid binding site required for infection. PLoS Pathog. *8*, e1002738.

Neu, U., Khan, Z.M., Schuch, B., Palma, A.S., Liu, Y., Pawlita, M., Feizi, T., and Stehle, T. (2013b). Structures of B-lymphotropic polyomavirus VP1 in complex with oligosaccharide ligands. PLoS Pathog. *9*, e1003714.

Neu, U., Maginnis, M.S., Palma, A.S., Ströh, L.J., Nelson, C.D.S., Feizi, T., Atwood, W.J., and Stehle, T. (2010). Structure-function analysis of the human JC polyomavirus establishes the LSTc pentasaccharide as a functional receptor motif. Cell Host Microbe *8*, 309–319.

Neu, U., Woellner, K., Gauglitz, G., and Stehle, T. (2008). Structural basis of GM1 ganglioside recognition by simian virus 40. Proc. Natl. Acad. Sci. USA *105*, 5219–5224.

Nilsson, J., Miyazaki, N., Xing, L., Wu, B., Hammar, L., Li, T.C., Takeda, N., Miyamura, T., and Cheng, R.H. (2005). Structure and assembly of a T=1 virus-like particle in BK polyomavirus. J. Virol. *79*, 5337–5345.

O'Donnell, J., Taylor, K.A., and Chapman, M.S. (2009). Adeno-associated virus-2 and its primary cellular receptor – cryo-EM structure of a heparin complex. Virology *385*, 434–443.

Papadimitriou, J.C., Randhawa, P., Rinaldo, C.H., Drachenberg, C.B., Alexiev, B., and Hirsch, H.H. (2016). BK polyomavirus infection and renourinary tumorigenesis. Am. J. Transplant. *16*, 398–406.

Pastrana, D.V., Ray, U., Magaldi, T.G., Schowalter, R.M., Cuburu, N., and Buck, C.B. (2013). BK polyomavirus genotypes represent distinct serotypes with distinct entry tropism. J. Virol. *87*, 10105–10113.

Pettersen, E.F., Goddard, T.D., Huang, C.C., Couch, G.S., Greenblatt, D.M., Meng, E.C., and Ferrin, T.E. (2004). UCSF Chimera – a visualization system for exploratory research and analysis. J. Comput. Chem. *25*, 1605–1612.

Ramos, E., Drachenberg, C.B., Wali, R., and Hirsch, H.H. (2009). The decade of polyomavirus BK-associated nephropathy: state of affairs. Transplantation *87*, 621–630.

Randhawa, P., Viscidi, R., Carter, J.J., Galloway, D.A., Culp, T.D., Huang, C., Ramaswami, B., and Christensen, N.D. (2009). Identification of species-specific and cross-reactive epitopes in human polyomavirus capsids using monoclonal antibodies. J. Gen. Virol. *90*, 634–639.

Rayment, I., Baker, T.S., Caspar, D.L., and Murakami, W.T. (1982). Polyoma virus capsid structure at 22.5 A resolution. Nature *295*, 110–115.

Schelhaas, M., Malmström, J., Pelkmans, L., Haugstetter, J., Ellgaard, L., Grünewald, K., and Helenius, A. (2007). Simian virus 40 depends on ER protein folding and quality control factors for entry into host cells. Cell *131*, 516–529.

Scheres, S.H.W. (2012). RELION: implementation of a Bayesian approach to cryo-EM structure determination. J. Struct. Biol. *180*, 519–530.

Schowalter, R.M., and Buck, C.B. (2013). The Merkel cell polyomavirus minor capsid protein. PLoS Pathog. *9*, e1003558.

Shen, P.S., Enderlein, D., Nelson, C.D.S., Carter, W.S., Kawano, M., Xing, L., Swenson, R.D., Olson, N.H., Baker, T.S., Cheng, R.H., et al. (2011). The structure of avian polyomavirus reveals variably sized capsids, non-conserved inter-capsomere interactions, and a possible location of the minor capsid protein VP4. Virology *411*, 142–152.

Stehle, T., Gamblin, S.J., Yan, Y., and Harrison, S.C. (1996). The structure of simian virus 40 refined at 3.1 A resolution. Structure *4*, 165–182.

Stehle, T., and Harrison, S.C. (1997). High-resolution structure of a polyomavirus VP1-oligosaccharide complex: implications for assembly and receptor binding. EMBO J. *16*, 5139–5148.

Stehle, T., and Harrison, S.C. (1996). Crystal structures of murine polyomavirus in complex with straight-chain and branched-chain sialyloligosaccharide receptor fragments. Structure *4*, 183–194.

Stolt, A., Sasnauskas, K., Koskela, P., Lehtinen, M., and Dillner, J. (2003). Seroepidemiology of the human polyomaviruses. J. Gen. Virol. *84*, 1499–1504.

Tillou, X., and Doerfler, A. (2014). Urological tumors in renal transplantation. Minerva Urol. Nefrol. *66*, 57–67.

Van der Aa, F., Beckley, I., and de Ridder, D. (2014). Polyomavirus BK – a potential new therapeutic target for painful bladder syndrome/interstitial cystitis? Med. Hypotheses *83*, 317–320.

Varki, A., Cummings, R.D., Aebi, M., Packer, N.H., Seeberger, P.H., Esko, J.D., Stanley, P., Hart, G., Darvill, A., Kinoshita, T., et al. (2015). Symbol nomenclature for graphical representations of glycans. Glycobiology *25*, 1323–1324.

Winter, B.J., O'Connell, H.E., Bowden, S., Carey, M., and Eisen, D.P. (2015). A case control study reveals that polyomaviruria is significantly associated with interstitial cystitis and vesical ulceration. PLoS One *10*, e0137310.

Zhang, K. (2016). Gctf: real-time CTF determination and correction. J. Struct. Biol. *193*, 1–12.

Zheng, S.Q., Palovcak, E., Armache, J.-P., Verba, K.A., Cheng, Y., and Agard, D.A. (2017). MotionCor2: anisotropic correction of beam-induced motion for improved cryo-electron microscopy. Nat. Methods *14*, 331–332.

STAR★METHODS

KEY RESOURCES TABLE

REAGENT or RESOURCE	SOURCE	IDENTIFIER
Bacterial and Virus Strains		
PGEM7-Dunlop (BK virus Dunlop strain)	Laboratory of Michael Imperiale, University of Michigan	N/A
Chemicals, Peptides, and Recombinant Proteins		
GT1b oligosaccharide	CarboSynth	Cat# OO16323
Heparin	Sigma	Cat# 4784
Deposited Data		
Density map of BKV in complex with GT1b	This paper	Electron Microscopy Data Bank: EMD-3944
Density map of BKV in complex with heparin	This paper	Electron Microscopy Data Bank: EMD-3945
Density map of unliganded BKV	This paper	Electron Microscopy Data Bank: EMD-3946
Density map of reduced BKV	This paper	Electron Microscopy Data Bank: EMD-4212
Coordinates for the BKV-GT1b asymmetric unit	This paper	PDB: 6ESB
Experimental Models: Cell Lines		
Vero cells	ATCC	CCL-81 RRID: CVCL_0059
Software and Algorithms		
RELION 2.0	(Kimanius et al., 2016; Scheres, 2012)	https://www2.mrc-lmb.cam.ac.uk/relion
MOTIONCOR2	(Zheng et al., 2017)	http://msg.ucsf.edu/em/software/motioncor2.html
gCTF	(Zhang, 2016)	https://www.mrc-lmb.cam.ac.uk/kzhang/Gctf/
UCSF Chimera	(Pettersen et al., 2004)	https://www.cgl.ucsf.edu/chimera/ RRID: SCR_004097
Coot	(Emsley et al., 2010)	https://www2.mrc-lmb.cam.ac.uk/personal/pemsley/coot/ RRID: SCR_014222
Phenix	(Headd et al., 2012)	https://www.phenix-online.org/ RRID: SCR_014224
PDBePISA	(Krissinel and Henrick, 2007)	http://www.ebi.ac.uk/pdbe/pisa/ RRID: SCR_015749
PyMOL	(The PyMOL Molecular Graphics System, Version 2.0, Schrödinger, LLC)	https://pymol.org/ RRID: SCR_000305

CONTACT FOR REAGENT AND RESOURCE SHARING

Further information and requests for resources and reagents should be directed to, and will be fulfilled by, the lead contact, Neil A. Ranson (n.a.ranson@leeds.ac.uk).

METHOD DETAILS

Cell Culture

Vero cells were maintained in DMEM with 10% fetal bovine serum (FBS) and 50 U/ml penicillin and streptomycin. Cells were grown at 37°C with 5% CO_2 in a humidified incubator.

Virus Growth

The genome of BKV Dunlop strain (a kind gift from Professor Michael Imperiale, University of Michigan) was prepared as reported previously (Abend et al., 2007). Briefly, the genome was excised from the pGEM7 Dunlop plasmid by *Bam*HI (New England Biolabs) digestion and then recircularized using T4 Ligase (New England Biolabs). Vero cells were seeded into 4 x T75 flasks and transfected with 4 μg DNA using NanoJuice (Novagen) according to the manufacturer's instructions with a DNA to Core ratio of 1:2 and DNA to Booster ratio of 1:3. Transfection complexes were removed 16 hr post transfection and replaced with fresh growth media. Ten days post transfection the cells were harvested by scraping into the media and subjected to three rounds of freeze-thaw using liquid N_2. The crude virus stocks were used to infect 36 x T175 flasks of 50% confluent Vero cells at 37°C; after 2 hr the virus was removed and replaced with fresh growth media.

Virus Purification

BKV-infected Vero cells were harvested 21 days post infection and purified essentially as described previously (Shen et al., 2011). Cells were harvested by scraping and pelleted at 4000 x *g* before being resuspended in 10 ml of buffer A (10 mM Tris, 50 mM NaCl, 0.01% Triton X-100) supplemented with an EDTA-free protease inhibitor cocktail (Roche). The lysate was freeze thawed 3 times with 3 min sonication in a water bath between each cycle. Deoxycholic acid was added to a final concentration of 0.25 % and incubated for 30 minutes at 37°C with shaking. The pH was then lowered to 6.0 with 0.5 M HEPES (pH 5.4) and 5 units of type V neuraminidase (Sigma) was added. This was incubated at 37°C for 1 hour with shaking and the pH was raised back to 7.5 with 0.5 M HEPES (pH 8.0). The sample was then sonicated for 3 x 45 seconds in a water bath before pelleting the cellular debris at 4000 x *g* for 10 minutes. The pellet was resuspended in 5 ml of buffer A and this process was repeated a further two times. The supernatants were combined over a 4 ml 20 % (w/v) sucrose cushion in buffer A before centrifugation at 85,000 x *g* for 3 hours at 4°C in a SW32Ti rotor (Beckman). The pellet was resuspended in 5 ml of 1.34 g/ml CsCl and the isopycnic gradient was spun at 4°C for 16 hours at 110,000 x *g* (no brake) in a SW55Ti rotor (Beckman). The BKV band was collected using a 26-gauge needle and dialysed against 2 L of buffer A (without Triton X-100) for 2 days at 4°C. Dialysis buffer was exchanged twice with pre-chilled buffer A (without Triton X-100).

Electron Microscopy

Cryo-EM grids were prepared by applying 3 μl of purified BKV (0.25 mg/ml) to 400 mesh lacey grids coated in a 3 nm carbon film (Agar Scientific, UK). The sample was left to adsorb for 30 seconds before most of the sample was blotted away manually and this process was repeated 4 times. For the reduced BKV structure, 5 mM DTT was added to the sample and incubated for 15 minutes at RT prior to preparing cryo-grids. On-grid binding of receptors was performed by applying 3 μl of a 20 mM solution of GT1b oligosaccharide (CarboSynth) or heparin 10 mg/ml (Sigma – 4784) in Buffer A (without Triton X-100) to the pre-blotted, BKV-coated grid, and leaving for 30 seconds before blotting and freezing using a Leica EM GP plunge freeze device (Leica Microsystems). The Leica EM chamber temperature was set to 8°C with 80 % relative humidity and liquid ethane was used for sample vitrification. Grids were glow discharged for 30 seconds prior to application of the samples. All data sets were collected on an FEI Titan Krios (ABSL, University of Leeds) transmission electron microscope at 300 kV, at a magnification of 75,000x and a final calibrated object sampling of 1.065 Å/pixel. Exposures were recorded using the EPU automated acquisition software on a FEI Falcon II (unliganded BKV) or Falcon III direct electron detector operating in linear mode. Detailed information on data collection is shown in Table S1.

Image Processing

Image processing was carried out using the RELION 2.0 pipeline (Kimanius et al., 2016; Scheres, 2012). Drift-corrected averages of each movie were created using MOTIONCOR2 (Zheng et al., 2017) and the contrast transfer function of each determined using gCTF (Zhang, 2016). Automated particle picking on lacey carbon grids resulted in a large number of boxes picked on the edges of holes in the carbon film. To remove such 'junk' particles from the data sets, 2D classification in RELION was used with CTF amplitude correction only performed from the first peak of each CTF onwards. Particles were further classified using several rounds of both reference-free 2D classification and 3D classification. After each round, the best classes/class was taken to the next step of classification. Icosahedral symmetry was imposed during 3D auto-refinement and post-processing was employed to appropriately mask the model, estimate and correct for the B-factor of the maps. The final resolutions were determined using the 'gold standard' Fourier shell correlation criterion (FSC = 0.143) shown in Table S1. Local resolution was estimated in RELION which also generated maps filtered by local resolution.

Model Building and Refinement

A preliminary model for the BKV asymmetric unit was generated by rigid body fitting our previously published homology model (5FUA) (Hurdiss et al., 2016) into the EM density map using UCSF Chimera (Pettersen et al., 2004). The model was then manually fitted in Coot using the 'real space refinement tool' (Emsley et al., 2010). The resulting model was then symmetrized in Chimera to generate the capsid and subject to refinement in Phenix (Headd et al., 2012). Iterative rounds of manual fitting in Coot and refinement in Phenix were carried out to improve non-ideal rotamers, bond angles and Ramachandran outliers. For VP2, the murine polyomavirus coordinates (1CN3) were aligned to a fivefold coordinated penton of BKV using UCSF Chimera. The VP2 model was then mutated, fitted and refined in Coot before being subject to refinement in Phenix. For GT1b modelling, the coordinates for the oligosaccharide component from the previously determined BKV penton – GD3 structure (Neu et al., 2013a) were used to model the disialic acid motif from the 'right' arm of GT1b. Low-pass filtered maps were generated using RELION image handler and difference maps were generated in UCSF Chimera. Interactions between chains 6 and 3 were analyzed using PDBePISA (Krissinel and Henrick, 2007). Figures were generated using UCSF Chimera and PyMOL (The PyMOL Molecular Graphics System, Version 2.0 Schrödinger, LLC).

Molecular Dynamics Simulations

The x-ray crystal structure 4MJ0 (Neu et al., 2013a), was used as a starting structure for MD. Missing protein loops were modelled with YASARA (Krieger and Vriend, 2015). The GT1b oligosaccharide was built with AMBER tleap using Glycam06 building blocks (Kirschner et al., 2008). The glycosidic torsions of the (2-8)-linkage were adjusted to the values found in the x-ray structure for GD3 in site C. GT1b molecules were positioned into the four occupied binding sites by superimposing the terminal Neu5Ac (R) with the terminal Neu5Acs present in the x-ray structure, respectively. An additional 5th GT1b molecule was manually positioned into the remaining binding site resembling the same binding mode. During very long MD simulations, dissociation of ligands can

occur, particularly when the binding mode is not optimized at the beginning of the simulation as is the case for 'docked ligands'. Since the binding mode of the terminal Neu5Ac (R) is well established by several H-bonds, distance restraints (Asn-272:OD1-Neu5Ac:N5, Ser-274:OG-Neu5Ac:O1A and Thr-276:OG1-Neu5Ac:O1B) were applied in order to guarantee that this residue stayed in the binding site in a defined binding mode for the whole simulation. A periodic box was filled with a 0.1% NaCl solution, which resulted in a molecular system consisting of 126,430 atoms. The simulations were performed at 310 K with YASARA in 'fast mode' using AMBER14 (which includes Glycam06) as a force field. In total 1.1 μs trajectory data was sampled for the BKV/GT1b pentamer complex distributed over six independent simulations. This allowed analysis of GT1b dynamics in the binding site(s) of BKV over an accumulated time of 5.5 μs. Snapshots were recorded every 50 ps, which gave a trajectory file consisting of 22,200 frames and produced about 20 GByte binary trajectory data in XTC format. All analysis and generation of scientific plots was performed with Conformational Analysis Tools (CAT) - www.md-simulations.de/CAT/. For calculation of the atom 3D density map all MD frames of the pentamer were superimposed on the EM atomic model using a sequence alignment file. In order to average the density over the five sides the five-fold symmetry was taken into account. Snapshots were visualized with VMD (Humphrey et al., 1996). Model coordinates are available from the authors upon request.

QUANTIFICATION AND STATISTICAL ANALYSIS

Where appropriate, statistical details are given in the Method Details section. Table S1 contains quantitative parameters related to data collection and image processing.

DATA AND SOFTWARE AVAILABILITY

The Cryo-EM maps of BKV-GT1b, BKV-heparin, unliganded-BKV and reduced-BKV reconstructions were deposited in the Electron Microscopy Data Bank under ID codes EMDB 3944, EMDB 3945, EMDB 3946 and EMDB 4212 respectively. The coordinates for the BKV-GT1b asymmetric unit were deposited in the Protein Data Bank under the ID code PDB 6ESB.

Structure, Volume *26*

Supplemental Information

The Structure of an Infectious Human Polyomavirus and Its Interactions with Cellular Receptors

Daniel L. Hurdiss, Martin Frank, Joseph S. Snowden, Andrew Macdonald, and Neil A. Ranson

Supplemental data items

Table S1 Data collection and image processing, related to STAR Methods.

	BKV-GT1b	BKV-heparin	BKV-unliganded	BKV-reduced
Microscope	FEI Titan Krios	FEI Titan Krios	FEI Titan Krios	FEI Titan Krios
Camera	Falcon III	Falcon III	Falcon II	Falcon III
Voltage (kV)	300	300	300	300
Pixel size (Å)	1.065	1.065	1.065	1.065
Total dose (e^-/Å^2)	59	55	40	66
Number of frames	59	39	39	45
Defocus range (µm)	-0.5 to -3.0	-0.5 to -2.3	-0.5 to -3.0	-0.5 to -3.0
Micrographs	8651	6227	3992	3474
Acquisition software	FEI EPU	FEI EPU	FEI EPU	FEI EPU
Motion correction	MotionCor2	MotionCor2	MotionCor2	MotionCor2
CTF estimation	Gctf	Gctf	Gctf	Gctf
Software	Relion2.0	Relion2.0	Relion2.0	Relion2.0
Particles contributed	40,334	24,791	11,980	8,733
B-factor	-212	-206	-174	-326
Resolution (FSC 0.143)	3.4	3.6	3.8	6.5

Supplemental Figures

Figure S1, related to Figure 1

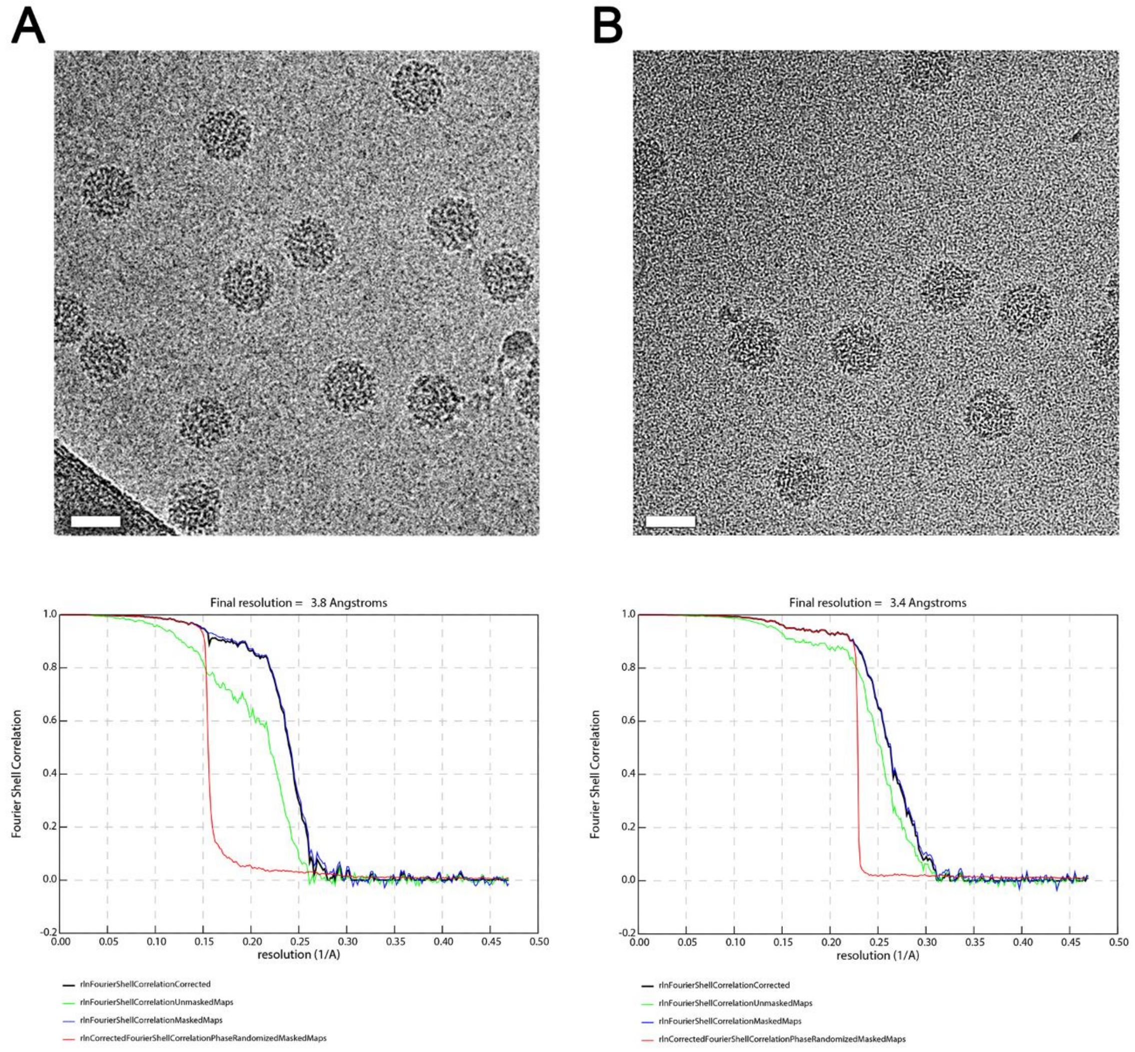

Typical micrographs from the BKV-unliganded (A) and BKV-GT1b (B) data sets (scale bar = 50 nm), with the plot of the corresponding Fourier shell correlation (FSC) below each. Based on the 0.143 criterion for the 'gold standard' comparison of two independent data sets, the resolution of the reconstructions is 3.8 Å (A) and 3.4 Å (B), respectively.

Figure S2, related to Figure 1

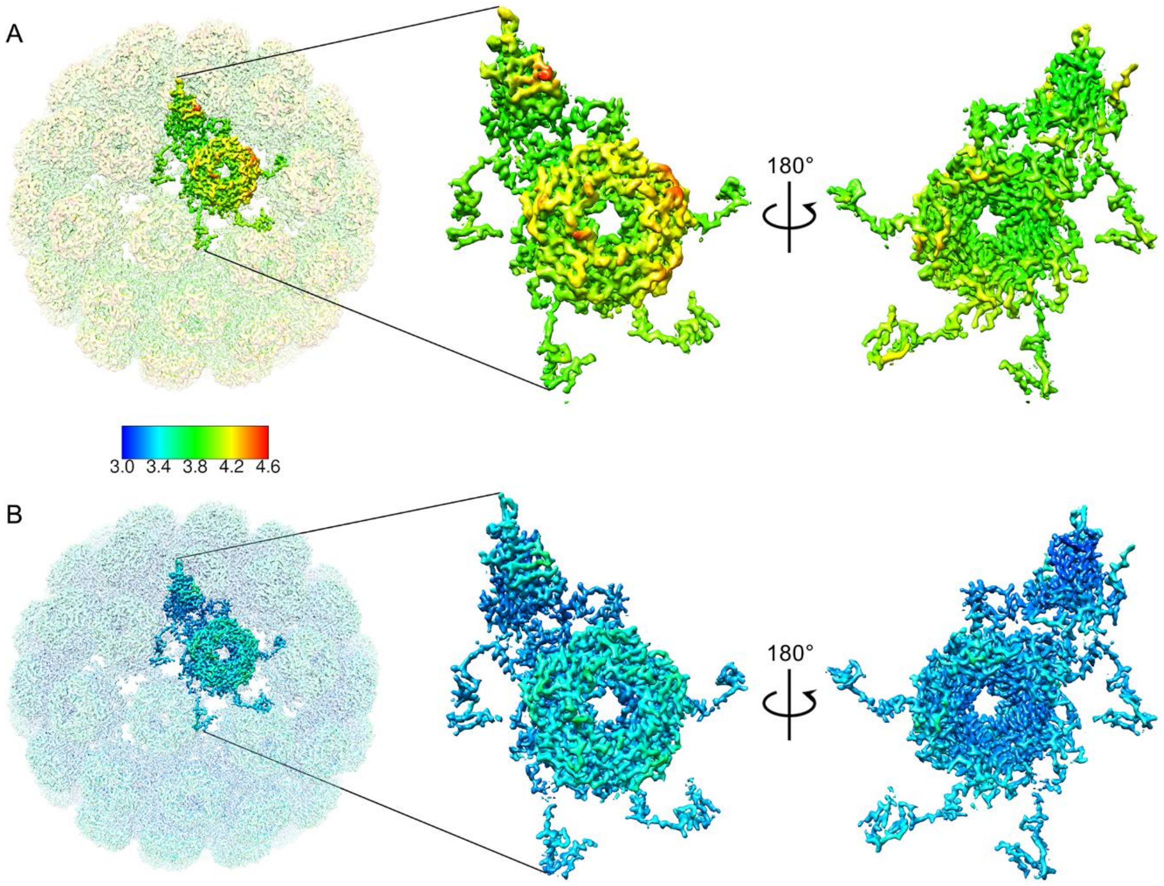

Local resolution of the BKV-unliganded (A) and BKV-GT1b (B) structures. A single asymmetric unit of BKV (seen from outside the capsid) is shown coloured according to its local resolution in both cases. The interior of the asymmetric unit is also shown by rotating the asymmetric unit 180° about the *y*-axis. The highest resolution bin is 3.0 Å (blue) and the lowest resolution bin is 4.6 Å (red). A key is shown for reference.

Figure S3, related to Figure 4

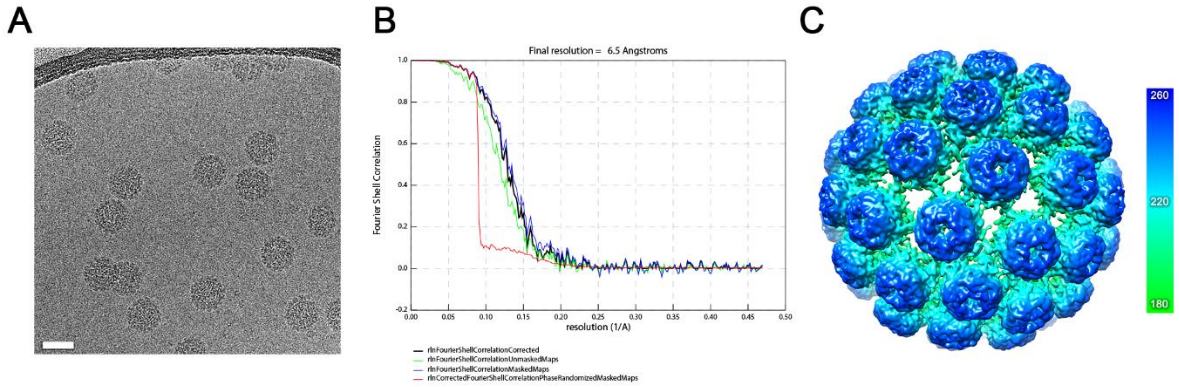

(A) Typical micrograph from the BKV-reduced data set (scale bar = 50 nm). (B) The plot of the Fourier shell correlation (FSC). Based on the 0.143 criterion for the 'gold standard' comparison of two independent data sets, the resolution of the reconstruction is 6.5 Å. (C) The 6.5 Å isosurface representation of reduced BKV viewed down an icosahedral two-fold axis (coloured according to the radial colouring scheme shown (Å)).

Figure S4, related to Figure 5

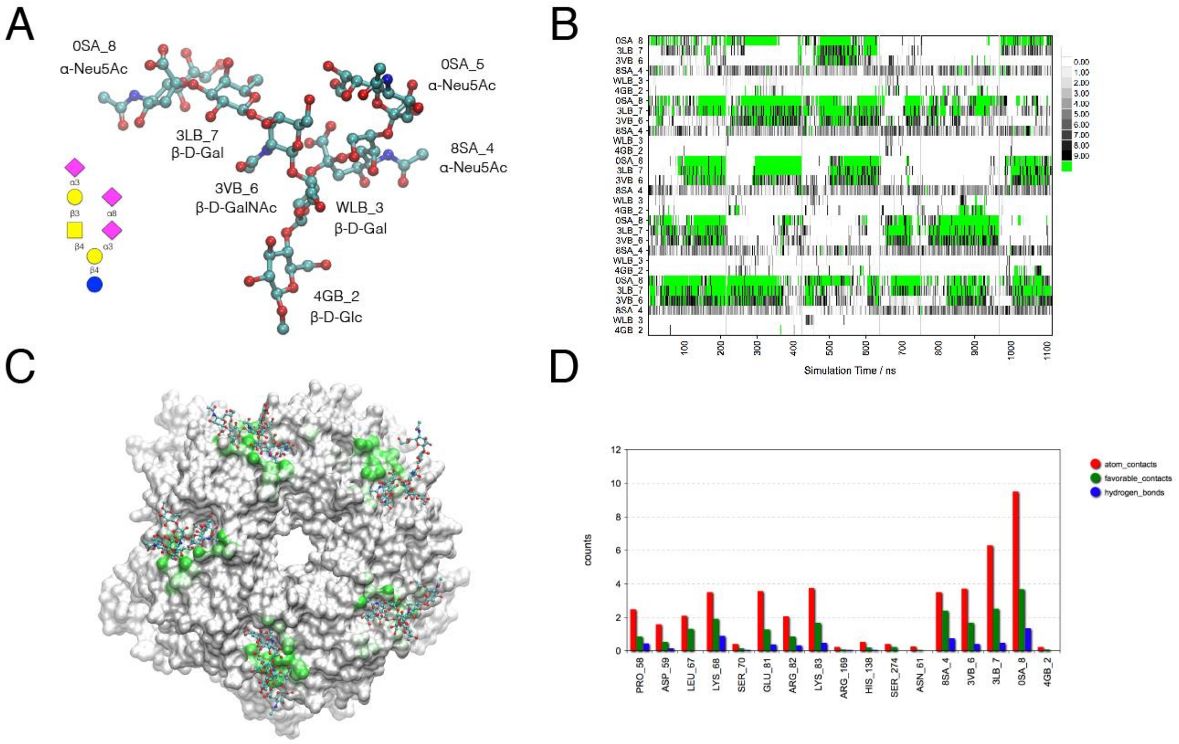

(A) SNFG-representation and 3D-model with Glycam residue labels of GT1b. (B) Trajectory of number of atom-atom contacts per residue for the five GT1B molecules. This shows the dynamics of the transient interactions. The various MD runs are separated by vertical lines. (C) Interaction 'footprint'. (D) Average number of atom-atom contacts for each residue significantly involved in binding. Note: Neu5Ac (R) (Glycam residue label 0SA_5) was excluded from the analysis.

Figure S5, related to Figure 5

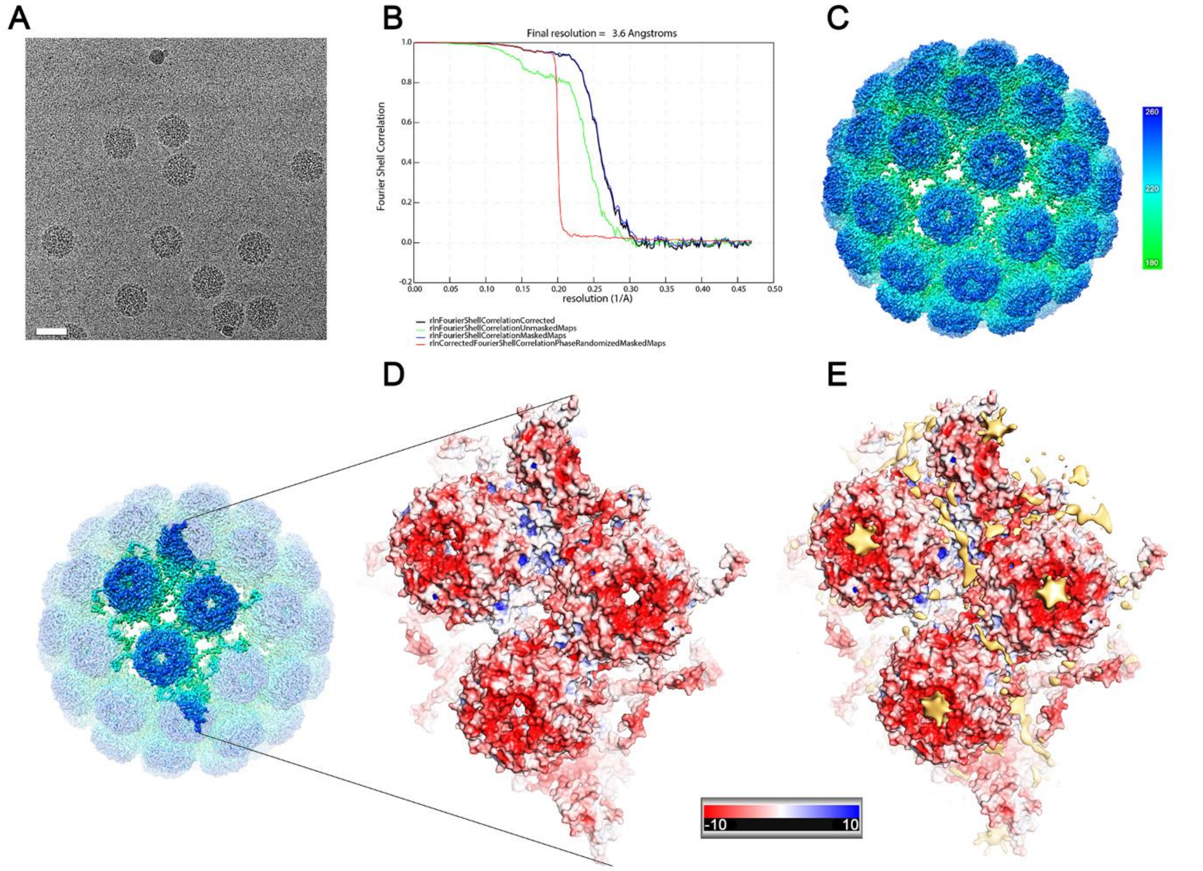

(A) Typical micrograph from the BKV-heparin data set (scale bar = 50 nm). (B) The plot of the Fourier shell correlation (FSC). Based on the 0.143 criterion for the 'gold standard' comparison of two independent data sets, the resolution of the reconstruction is 3.6 Å. (C) An isosurface representation of the 3.6 Å structure of BKV (coloured according to the radial colouring scheme shown (Å)). (D) Enlarged section of the BKV capsid with the surface colored according to electrostatic surface potential (Baker et al., 2001). (E) The same view shown in (D) overlaid with the putative heparin difference density shown in Figure 5H (orange).
